# Spatial patterns of progression in reported glioblastoma cohorts after upfront chemoradiation and salvage therapy: a systematic review and meta-analysis

**DOI:** 10.1007/s11060-026-05734-w

**Published:** 2026-07-30

**Authors:** Rafal Chojak, Noah B. Drewes, Katarzyna Slychan, Rimas V. Lukas

**Affiliations:** 1 Department of Neurological Surgery, Feinberg School of Medicine, Northwestern University, 676 N St. Clair St, Suite 2210, Chicago, IL 60611 USA; 2 Malnati Brain Tumor Institute of the Robert H. Lurie Comprehensive Cancer Center, Feinberg School of Medicine, Northwestern University, Chicago, IL 60611 USA; 3 Department of Neurosurgery, St Lucas Hospital, Tarnow, Poland; 4 Department of Neurology, Northwestern University Feinberg School of Medicine, Chicago, IL USA

**Keywords:** Glioblastoma, Patterns of failure, Recurrence, Chemoradiation, Salvage therapy

## Abstract

**Purpose:**

Spatial patterns of glioblastoma (GBM) progression inform focal salvage eligibility, but interpretation across studies is limited by heterogeneous spatial definitions, high between-study heterogeneity, and incomplete reporting of non-enhancing/FLAIR-dominant progression. We synthesized reported progression-location involvement after upfront radiotherapy/temozolomide (RT/TMZ) and salvage therapy.

**Methods:**

We systematically searched PubMed and Scopus from January 1, 2000, to February 1, 2026, and performed arm-level random-effects meta-analysis of logit-transformed proportions. Enhancing progression was harmonized as non-mutually exclusive local/in-field, marginal/field-edge, and distant/out-of-field involvement among evaluable cases, using each study’s spatial framework. The supplementary broad escape-pattern construct was defined as reported progression beyond purely local/in-field enhancing failure.

**Results:**

We included 107 studies (122 arms; 10,529 patients). At first progression after upfront RT/TMZ ± TTF, reported local/in-field enhancing involvement among evaluable cases was 79.2% (95% CI 75.7–82.3; k = 94; *n* = 6,989; I²=84.8%), and distant/out-of-field involvement was 16.9% (95% CI 14.3–19.9; k = 89; *n* = 6,493; I²=85.2%). After salvage therapy, local/in-field involvement remained the majority pattern reported among evaluable cases (59.8%, 95% CI 52.8–66.4; k = 27; *n* = 1,572; I²=80.5%), with distant/out-of-field involvement of 17.5% (95% CI 12.9–23.3; k = 21; *n* = 1,307; I²=75.5%) and a supplementary broad escape-pattern estimate of 38.6% (95% CI 31.9–45.9; k = 27; *n* = 1,572; I²=80.1%). Non-enhancing/FLAIR-dominant progression was sparsely and likely selectively reported; only six recurrent/salvage arms contributed data, yielding an exploratory pooled estimate of 27.8% (95% CI 12.8–50.3; *n* = 216; I²=75.7%).

**Conclusion:**

Published GBM cohorts show predominantly local/in-field enhancing progression after upfront therapy (~ 80%) and persistent majority local/in-field involvement after salvage therapy (~ 60%). Standardized reporting of enhancing, non-enhancing/FLAIR-dominant, disseminated, posterior fossa, and brainstem progression is needed.

**Supplementary Information:**

The online version contains supplementary material available at https://doi.org/10.1007/s11060-026-05734-w.

## Introduction

Adult glioblastoma almost invariably progresses despite maximal safe resection, focal radiotherapy, temozolomide, and, in selected patients, tumor-treating fields, and survival after recurrence remains limited despite available salvage therapies [1–7]. The spatial pattern of first progression - confined to the pretreated bed, adjacent to the high-dose field edge, out-of-field/distant, multifocal/disseminated, or predominantly non-enhancing on T2/FLAIR - directly constrains salvage options and trial eligibility. Focal approaches are generally most feasible when progression remains localized, whereas marginal/field-edge, distant/out-of-field, multifocal, and non-enhancing/FLAIR-dominant progression can complicate target definition, normal-tissue constraints, and interpretation of focal salvage outcomes [3, 8–13].

Studies conducted in the modern chemoradiotherapy era have generally suggested that first progression after upfront RT/TMZ occurs predominantly within or near the original high-dose treatment region, supporting contemporary target-volume strategies and the expectation that many early failures remain spatially localized [14–16]. However, reported estimates vary substantially across studies. Even among upfront RT/TMZ-treated cohorts, the proportion of patients with local or in-field progression has ranged from approximately one-half to more than 90%, while distant progression has ranged from uncommon to a substantial minority or even majority of events in selected series [8–11, 17–25].

The problem is amplified in the recurrent and salvage-treated setting. Clinically, progression after successive therapies is often perceived as more spatially distributed, multifocal, and/or non-enhancing, but the extent to which this reflects treatment-driven biology, selection of patients who survive long enough to receive later-line therapy, or differences in imaging ascertainment remains uncertain [18, 23, 26, 27]. Bevacizumab is particularly relevant because it can reduce vascular permeability and suppress contrast enhancement, producing radiographic “pseudoresponse” and potentially decoupling enhancement from viable tumor burden [10, 18, 26, 27]. As a result, assessment after bevacizumab or other salvage therapies may depend more heavily on T2/FLAIR abnormalities, yet non-enhancing or FLAIR-dominant progression is inconsistently defined, inconsistently enumerated, and rarely reported as a distinct mutually exclusive spatial endpoint [10, 18, 26, 27]. These limitations complicate interpretation of recurrence-pattern studies and weaken the ability to compare upfront and salvage-treated disease.

To address these gaps, we performed a systematic review and arm-level meta-analysis of spatial progression patterns in GBM at two clinically distinct time points: (i) first progression after upfront RT/TMZ with or without tumor-treating fields and (ii) subsequent progression after a defined salvage therapy line. Our aim was to describe reported involvement patterns across a heterogeneous literature, quantify between-study variability, and identify reporting deficiencies that limit interpretation. Specifically, we sought to harmonize reported spatial categories, quantify local versus broader spatial progression in upfront and salvage-treated settings, and assess how often non-enhancing or FLAIR-dominant progression is explicitly reported. These findings may help contextualize focal salvage eligibility and trial design, but their primary contribution is to support standardized reporting of spatial involvement, denominator definitions, radiotherapy reference volumes, and non-enhancing/FLAIR-dominant progression in future studies.

## Methods

### Study design and reporting

We conducted a systematic review and meta-analysis to describe spatial patterns of radiographic progression in adult glioblastoma, aiming to summarize what is typically reported at first progression after upfront therapy and at subsequent progression after salvage therapy. The review was performed and reported in accordance with PRISMA 2020 and PRISMA-S. This review was not prospectively registered.

### Information sources and search strategy

We searched MEDLINE (PubMed) and Scopus from 1 January 2000 through 1 February 2026 using a strategy combining GBM recurrence/progression concepts with spatial or pattern-of-failure terminology. Database-specific strategies are provided in the Supplementary Table 1. We additionally screened reference lists of included studies and relevant reviews.

### Eligibility criteria

We included prospective trials, observational cohorts, and case series enrolling adults (≥ 18 years) with histologically or pathologically diagnosed glioblastoma as reported by the original investigators, using the terminology employed in the source publication, including glioblastoma, glioblastoma multiforme, WHO grade IV astrocytoma, or GBM NOS. Because the review covered a broad diagnostic era, diagnoses were not reclassified according to contemporary molecular criteria unless such information was explicitly reported. Consequently, some older cohorts labeled as GBM may have included tumors that would now be classified as IDH-mutant grade 4 astrocytoma or, less commonly, other adult diffuse glioma entities under contemporary WHO criteria; this diagnostic-era heterogeneity was captured when reported and considered in interpretation. Mixed high-grade glioma or malignant glioma cohorts were eligible only if GBM comprised at least 75% of the cohort or if GBM-specific spatial progression outcomes were extractable. Eligible studies were required to report extractable numerators and denominators for progression location using an explicit spatial classification scheme.

Studies were eligible if they reported spatial patterns for first progression after upfront radiotherapy with temozolomide, with or without tumor-treating fields, and/or spatial patterns for a defined salvage line before the indexed progression event, including re-irradiation, bevacizumab-containing therapy, and other systemic salvage regimens when reported. We excluded pediatric-only cohorts, studies focused exclusively on non-GBM or lower-grade gliomas, spinal gliomas, non-clinical or pathology-only studies, reports with fewer than 20 relevant patients, studies without sufficient detail to classify progression location or determine the relevant denominator for spatial outcomes and overlapping reports from the same indexed clinical setting. When overlapping reports were identified, we retained the most comprehensive dataset with the largest extractable denominator and most complete spatial-outcome reporting. Excluded or merged overlapping reports are listed in Supplementary Table 2.

## Study selection and data extraction

Three reviewers independently screened titles and abstracts and then evaluated full texts for eligibility. Disagreements were resolved by consensus. Data were extracted independently by three reviewers (RC, ND, KS), and discrepancies were resolved by consensus. Spatial harmonization decisions, including assignment of source-study categories to local/in-field, marginal/field-edge, distant/out-of-field, diffuse, nonlocal, and broad escape-pattern constructs, were independently checked by at least two reviewers. Difficult classifications were adjudicated by consensus with reference to the original study’s radiotherapy dose distribution, distance threshold, imaging definition, or anatomic description. Data were extracted at the arm level using a standardized form capturing study characteristics (publication year, geography, design), imaging cadence when reported, therapy context immediately preceding the indexed progression event, salvage modality category, and mixed-histology handling. We extracted counts and denominators for enhancing spatial involvement outcomes. During audit and harmonization, we additionally derived spatial reference framework, recurrence-pattern framework, the relation between the enhancing denominator and the total at-risk cohort, and overlap-cluster annotations used to retain only the most comprehensive dataset when same-setting cohort overlap was identified.

The unit of analysis for spatial proportions followed each study’s reporting framework. For patient-level reporting, denominators reflected the number of patients in the relevant arm who were evaluable for spatial classification as stated by the study (e.g., all treated, all progressors, or mapped/evaluable subsets). Prespecified data extraction rules are detailed in Supplementary Table 3; mapping of source-study spatial definitions to harmonized involvement categories are in Supplementary Table 4; study-specific mapping decisions and difficult classifications are provided in Supplementary Table 5.

### Spatial endpoints and involvement-based harmonization

The indexed endpoint was the first radiographic progression event reported for each therapy context within a study. Contexts were classified as first progression after upfront RT/TMZ ± TTF or subsequent progression after a defined salvage therapy line (often second progression, but not reliably distinguishable from third line and beyond when studies did not specify line). When re-irradiation was delivered with concurrent or peri-reRT bevacizumab, arms were classified by the primary salvage modality (reRT) and BEV co-administration was recorded when reported. Because most studies did not report mutually exclusive recurrence categories, enhancing progression was harmonized using a non-mutually exclusive involvement framework. Each study’s spatial scheme was mapped relative to the source study’s own reference framework, including dose-based volumes or isodose shells, distance-from-cavity/tumor thresholds, and anatomic descriptors. Local/in-field involvement captured progression within or contiguous with the original high-dose treatment region or primary site, as defined by the source study. Marginal/field-edge involvement captured adjacent, intermediate, partial-overlap, or field-edge recurrence categories. Distant/out-of-field involvement captured remote intracranial progression outside the relevant high-dose region or beyond the source study’s distance/anatomic threshold. When studies reported both local and distant components, events contributed to both locations; therefore, involvement proportions were not expected to sum to 100%. As a supplementary sensitivity construct, we defined broad escape-pattern progression as reported progression beyond purely local/in-field enhancing failure, using reported nonlocal involvement when available and otherwise a harmonized composite of marginal/field-edge, diffuse, multifocal/disseminated, and/or distant/out-of-field categories when those were the only extractable categories. Because ‘broad escape’ is not a universally standardized recurrence-pattern term, it was used only as a descriptive sensitivity construct and not as a replacement for local/in-field, marginal/field-edge, and distant/out-of-field reporting. Non-enhancing/FLAIR-dominant progression was extracted only when explicitly defined and enumerated with a clear numerator and denominator. In bevacizumab-exposed or other salvage cohorts, we recorded whether the source study used enhancement-only criteria, incorporated T2/FLAIR progression, or separately enumerated non-enhancing/FLAIR-dominant events.

### Outcomes

The primary outcomes were pooled estimates of reported local/in-field and distant/out-of-field enhancing involvement among evaluable cases at first progression after upfront RT/TMZ ± TTF and at progression after salvage therapy. Salvage analyses were additionally stratified by modality where eligible. Secondary outcomes were reported marginal/field-edge enhancing involvement and the supplementary broad escape-pattern construct. Non-enhancing/FLAIR-dominant progression in recurrent/salvage cohorts was assessed as an exploratory outcome when explicitly defined and quantitatively synthesizable.

### Risk of bias and reporting-quality assessment

Two reviewers independently assessed risk of bias and reporting quality using a structured, domain-based framework adapted from the Joanna Briggs Institute critical appraisal tools for prevalence studies and case series/cohort evidence. Because the outcome of interest was recurrence location, the assessment was tailored to factors most likely to bias prevalence or proportion estimates of spatial recurrence patterns. The evaluated domains included study design and sampling frame, completeness and consistency of imaging follow-up, clarity of progression criteria, reproducibility of spatial recurrence definitions, transparency of denominators, handling of non-enhancing or FLAIR-dominant progression, mixed-histology or diagnostic-era concerns, and potential overlap with other included reports. Disagreements were resolved by consensus. Studies were considered at higher risk of ascertainment or reporting bias when recurrence location was reported only for selectively imaged or spatially mapped patients, imaging schedules or follow-up completeness were unclear, spatial categories were ambiguous or not reproducible, non-enhancing progression was not addressed in salvage or bevacizumab-exposed cohorts, or denominators differed across endpoints without explanation.

### Statistical analysis

Study-level proportions were summarized using random-effects meta-analysis on the logit scale and back-transformed to the proportion scale for presentation. The primary model used a logit-transformed random-effects approach with restricted maximum likelihood estimation of between-study variance (REML τ²), a 0.5 continuity correction for studies with zero or complete events when required, and Hartung-Knapp adjustment for confidence intervals. Pooled estimates are reported with 95% confidence intervals, and 95% prediction intervals were calculated and back-transformed to the proportion scale when estimable to describe the expected range of reported involvement in comparable future studies. Heterogeneity was summarized using τ² and I².

Because outcomes were defined as involvement rather than mutually exclusive recurrence categories, each location was meta-analyzed separately using the denominator reported for that spatial assessment in the original study. Pooled estimates were interpreted as descriptive summaries of reported involvement among evaluable cases, not as absolute probabilities of a given failure pattern.

Prespecified descriptive stratification focused on therapy context and salvage modality. During final audit, potentially overlapping same-setting cohort reports were collapsed by retaining the most comprehensive dataset before meta-analysis. Univariable meta-regression analyses were performed as exploratory assessments of heterogeneity and should be interpreted descriptively. Small-study effects were assessed using funnel-plot asymmetry tests for endpoints with sufficient numbers of arms, and trim-and-fill analyses were used as sensitivity analyses rather than definitive tests of publication bias. Study-level and pooled results were displayed using forest plots and funnel plots. All analyses were conducted in RStudio, using the metafor package for random-effects models, heterogeneity estimates, prediction intervals, meta-regression, and small-study-effect analyses.

## Results

**Fig. 1:**
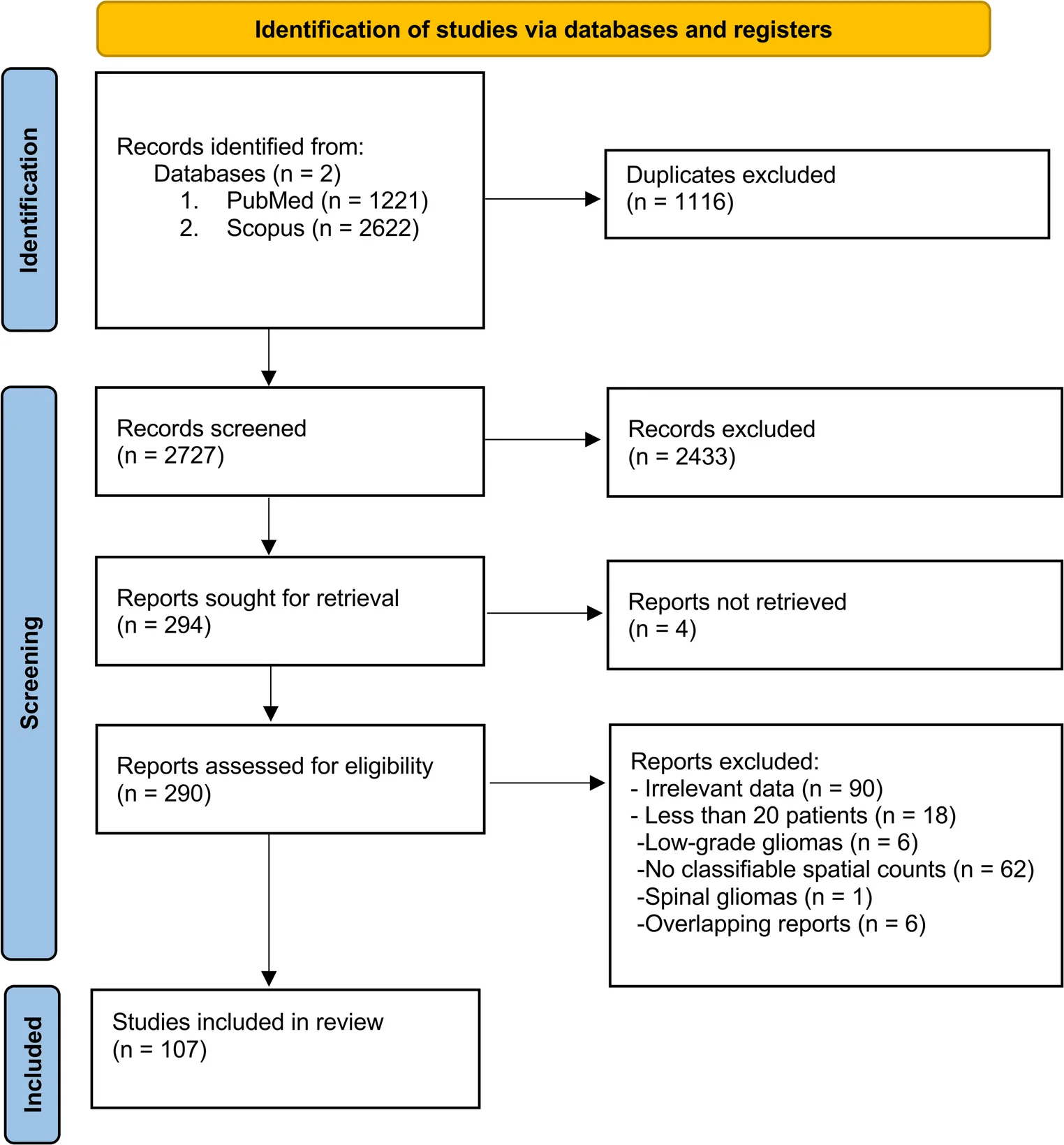
PRISMA flow diagram of study identification, screening, overlap adjudication, and quantitative inclusion

Database searches identified 3,843 records. After removing duplicates (*n* = 1,116), 2,727 records were screened and 2,433 were excluded. These title/abstract exclusions were records that clearly did not meet eligibility because they did not report adult intracranial GBM/GBM-dominant high-grade glioma spatial progression patterns, were non-clinical/basic-science reports, reviews/editorials/case reports, pediatric or non-brain glioma studies, treatment-outcome studies without extractable recurrence-location data, or otherwise lacked classifiable spatial progression counts. Of 294 reports sought for retrieval, 4 were not retrieved and 290 reports were assessed for eligibility. Of 290 reports assessed in full text, 107 studies [8, 18–24, 26–124] met inclusion criteria after overlap adjudication and were included in the quantitative synthesis (Fig. 1).

### Study and arm characteristics

After resolving overlapping same-setting cohorts by retaining the most comprehensive report, the analytic dataset comprised 122 extracted arms from 107 studies spanning 2003–2026 (median publication year 2017, IQR 2013–2022). Most arms were retrospective cohorts (73.8%), with fewer clinical trials (12.3%) and prospective cohorts (8.2%); the remainder were secondary/post hoc analyses of existing trial or prospective-study cohorts, or mixed designs. Arms were geographically diverse (North America 35.2%, Europe 34.4%, Asia 23.0%), most commonly from the United States (*n* = 36), Germany (*n* = 21), and Japan (*n* = 14). By therapy context, 95 arms represented first progression after upfront RT/TMZ ± TTF, and 27 arms represented progression after salvage therapy (post-BEV failure *n* = 12; post-reRT failure *n* = 13; post-other failure *n* = 2). Per-arm cohort size was moderate (median 56 patients, IQR 34–94; total *n* = 10,529 across arms). Imaging cadence was reported in 68.9% of arms (median 10 weeks, IQR 8–12); reported cadence was shorter after BEV failure (median 8 weeks) than after re-irradiation (median 12 weeks). Endpoint reporting was near-complete for local/in-field involvement (121/122) and high for distant/out-of-field involvement (110/122), while marginal/field-edge involvement was reported in 55/122 arms, consistent with selective reporting and variable operational definitions. Non-enhancing events were explicitly enumerated in 8/122 arms; marginal and broad escape-pattern summaries are therefore presented as supplementary analyses. Only a small minority of included sources were mixed high-grade glioma or malignant glioma cohorts (7/107 studies; 8/122 arms), all meeting prespecified GBM-dominant or GBM-extractable eligibility criteria.

### Risk-of-bias and reporting-quality summary

Two reviewers independently assessed risk of bias and reporting quality using an adapted Joanna Briggs Institute domain-based framework for prevalence/proportion and case-series/cohort evidence (Supplementary Table 6). Domains focused on factors most likely to bias spatial recurrence estimates, including sampling frame, imaging follow-up, progression criteria, spatial-definition reproducibility, denominator transparency, non-enhancing/FLAIR-dominant progression, mixed-histology or diagnostic-era concerns, and potential cohort overlap. Disagreements were resolved by consensus.

Using the adapted JBI prevalence framework, 30/122 arms were rated as low concern, 75/122 as moderate concern, and 17/122 as high concern. The most frequent limitations were unverifiable retrospective sampling or recruitment approach, endpoint-specific or incomplete evaluable denominators, nonstandard or unclear spatial classification, and incomplete imaging-cadence or follow-up reporting. No study was excluded on the basis of this appraisal; instead, ratings were used to contextualize certainty and interpretation of the pooled estimates.

Quality-based sensitivity analyses excluding study arms rated as high concern yielded materially unchanged pooled estimates. Across the main estimable upfront, post-bevacizumab, and post-reirradiation analyses, absolute changes in pooled estimates were small, generally within 1% point and no greater than 1.9% points.

### Pooled patterns of enhancing progression

**Fig. 2:**
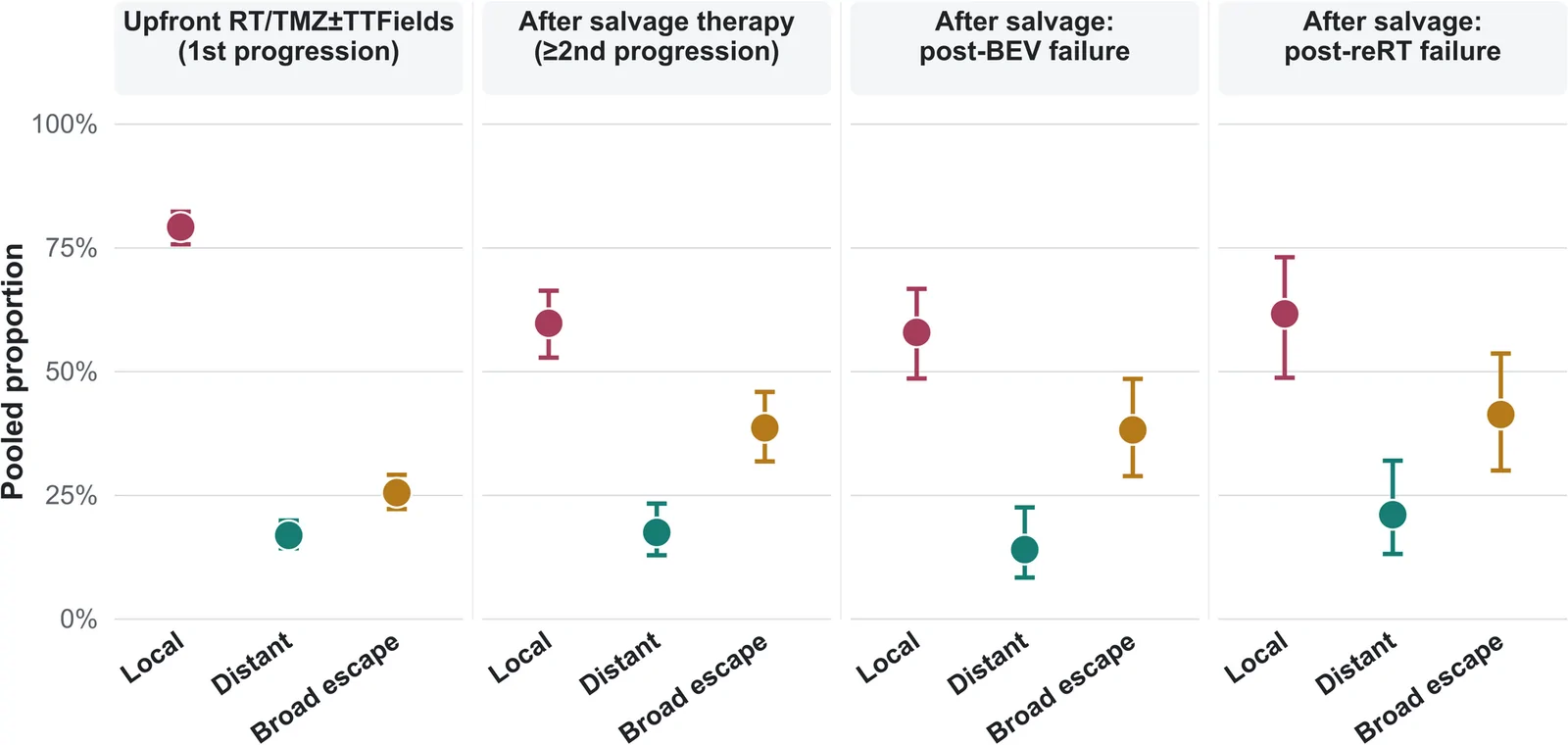
Pooled enhancing spatial involvement across upfront and salvage strata. Points and whiskers show random-effects estimates and 95% CIs for local/in-field, distant/out-of-field, and broad escape-pattern progression. Categories are non-mutually exclusive and may not sum to 100%. BEV, bevacizumab; reRT, re-irradiation; RT, radiotherapy; TMZ, temozolomide; TTF, tumor-treating fields

**Fig. 3:**
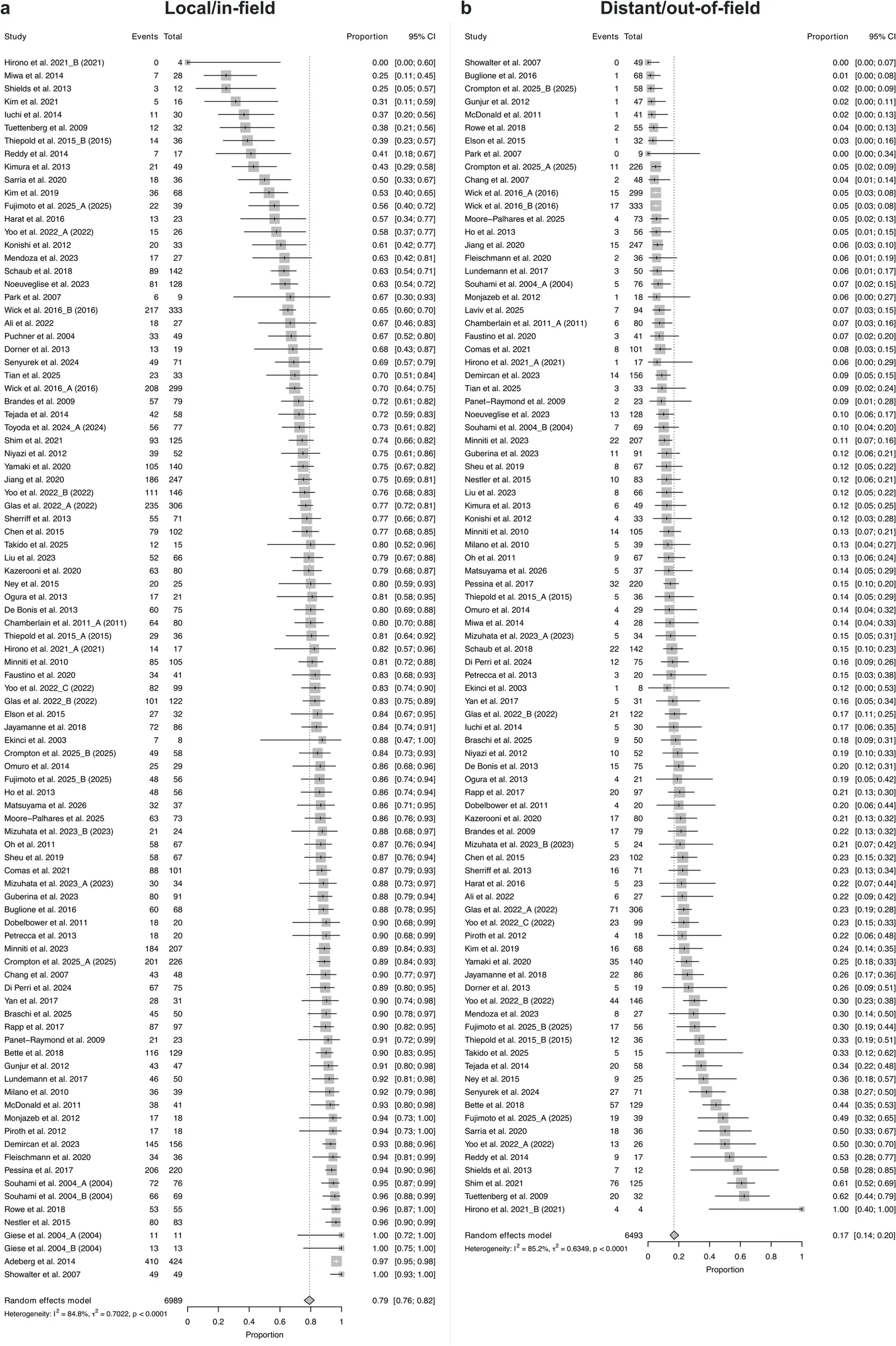
Random-effects forest plots of contrast-enhancing tumor involvement at first progression following upfront RT/TMZ ± TTF: (**a**) local/in-field involvement and (**b**) distant/out-of-field involvement. Squares represent study-specific proportions, with square size reflecting study weight; horizontal lines indicate 95% confidence intervals, and diamonds represent pooled random-effects estimates

Across upfront cohorts at first progression after RT/TMZ ± TTF, reported local/in-field enhancing involvement among evaluable cases was the dominant reported pattern (79.2%, 95% CI 75.7–82.3; k = 94 arms; *n* = 6,989; I²=84.8%; 95% PI, 41.6–95.3). Reported distant/out-of-field enhancing involvement among evaluable cases was 16.9% (95% CI 14.3–19.9; k = 89; *n* = 6,493; I²=85.2%; 95% PI, 4.0-50.1) (Figs. 2 and 3). Marginal enhancing involvement pooled at 10.3% (95% CI 7.9–13.4; k = 46; *n* = 3,003; 95% PI, 2.0-38.9), and broad escape-pattern progression pooled at 25.5% (95% CI 22.2–29.2; k = 89; *n* = 6,094; 95% PI, 7.4–59.4) (Supplementary Fig. 1).

**Fig. 4:**
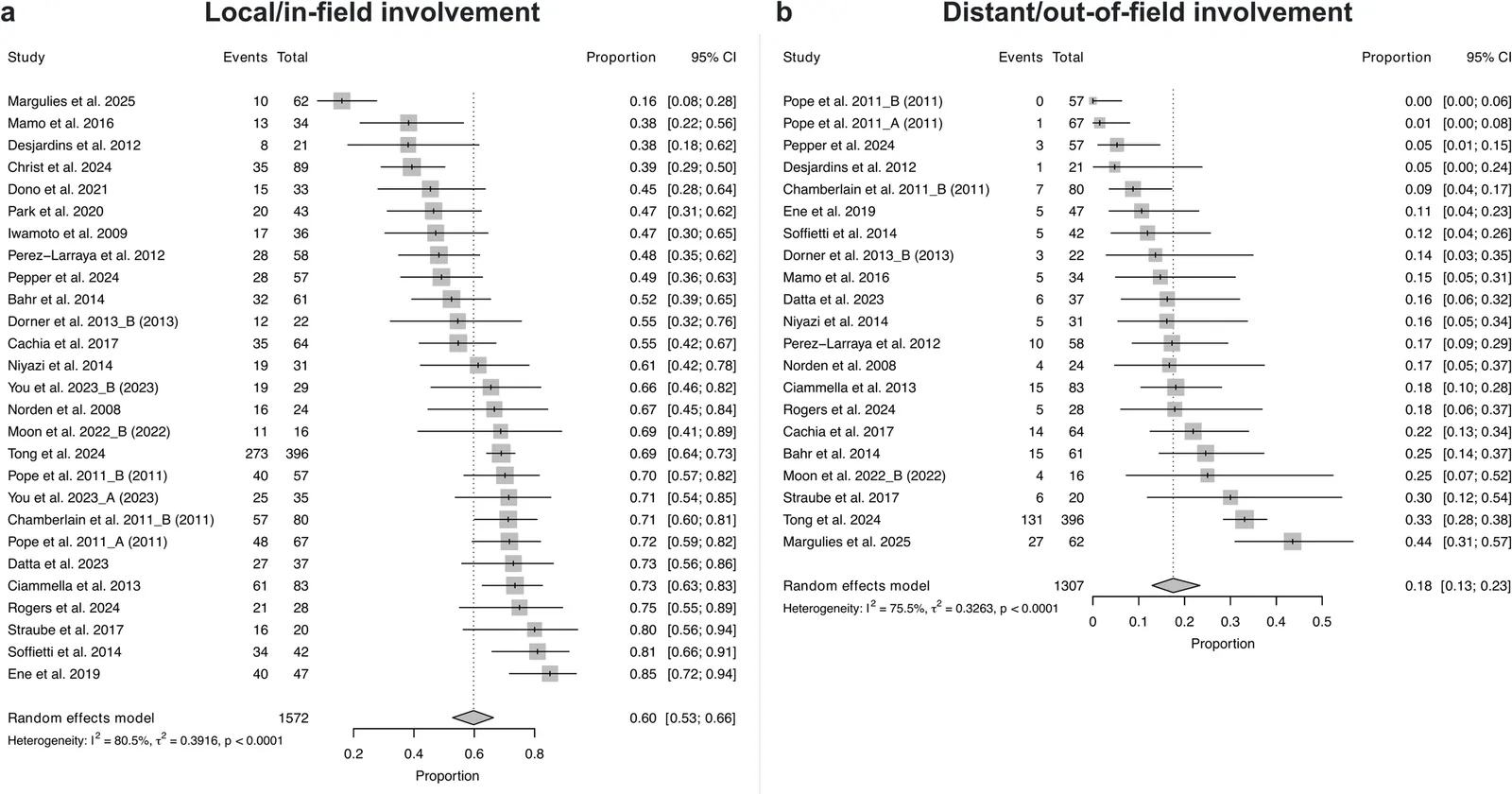
Random-effects forest plots of enhancing involvement at progression after salvage therapy: (**a**) local/in-field and (**b**) distant/out-of-field. Squares show study-specific proportions, horizontal lines indicate 95% confidence intervals, and diamonds represent pooled estimates

In salvage-treated cohorts at subsequent progression, reported local/in-field enhancing involvement remained the majority finding among evaluable cases (59.8%, 95% CI 52.8–66.4; k = 27; *n* = 1,572; I²=80.5%; 95% PI, 28.4–84.8), although the estimate was numerically lower than in upfront first-progression cohorts. Distant/out-of-field involvement pooled at 17.5% among evaluable cases (95% CI 12.9–23.3; k = 21; *n* = 1,307; I²=75.5%; 95% PI, 5.7–42.5) (Figs. 2 and 4). Marginal enhancing involvement pooled at 17.4% (95% CI 8.8–31.6; k = 9; *n* = 830; 95% PI, 1.9–69.7), and broad escape-pattern progression pooled at 38.6% (95% CI 31.9–45.9; k = 27; *n* = 1,572; 95% PI, 13.5–71.8) (Supplementary Fig. 2).

### Progression after salvage therapy subgroup analyses by salvage context

Within salvage-treated cohorts, reported local/in-field involvement among evaluable cases was 57.9% after BEV failure (95% CI 48.6–66.7; k = 12; *n* = 587; 95% PI, 30.4–81.3) and 61.7% after reRT failure (95% CI 48.8–73.1; k = 13; *n* = 947; 95% PI, 20.5–91.0). Reported distant/out-of-field involvement was 14.0% after BEV failure (95% CI 8.4–22.6; k = 10; *n* = 508; 95% PI, 4.6–35.8) and 21.1% after reRT failure (95% CI 13.2–32.0; k = 9; *n* = 761; 95% PI, 5.1–56.8). These arm-level estimates should not be interpreted as evidence that salvage modality itself changes progression location.

### Non-enhancing/FLAIR-dominant progression as a reporting gap

**Fig. 5:**
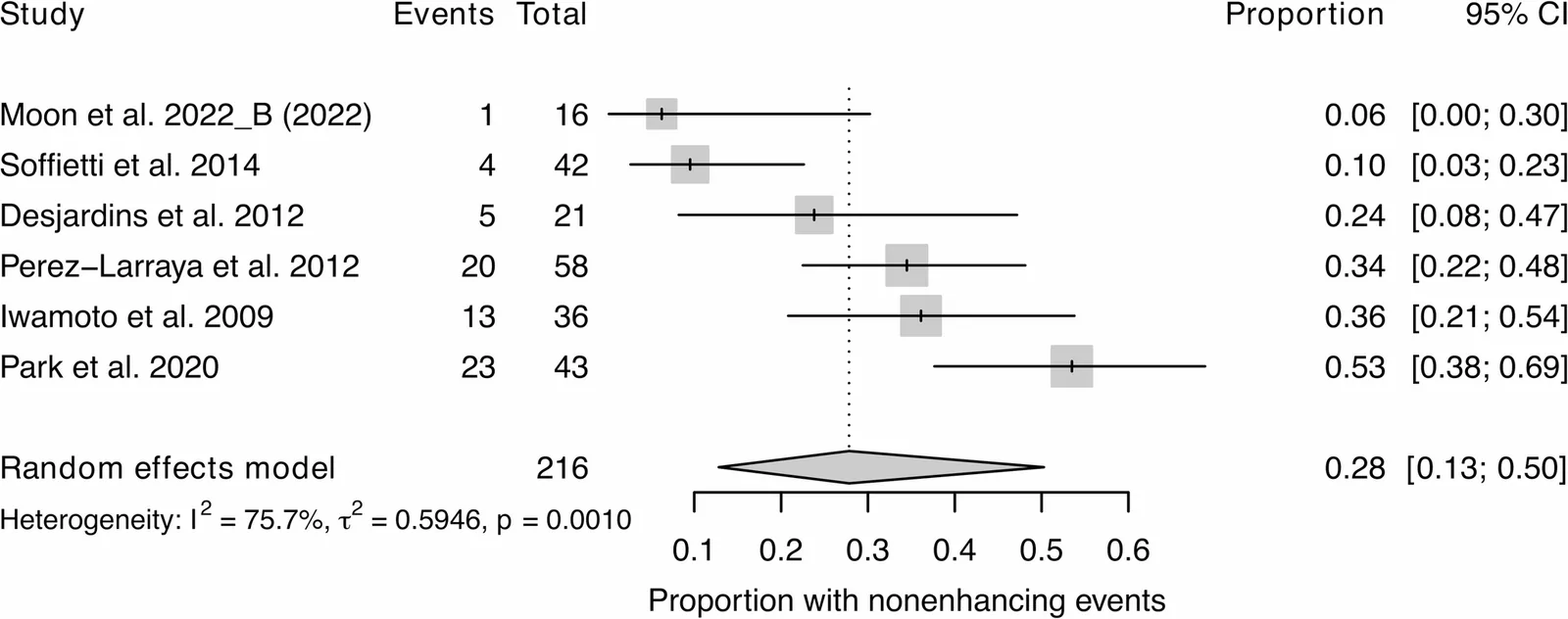
Random-effects forest plot of reported non-enhancing/FLAIR-dominant progression after salvage therapy. Squares represent study-specific proportions, horizontal lines indicate 95% confidence intervals, and the diamond shows the pooled estimate. FLAIR, fluid-attenuated inversion recovery

Reporting of non-enhancing (FLAIR-dominant) progression was sparse overall (7/107 studies, 6.5%) but more common in recurrent/salvage cohorts (6/25 studies, 24.0%). Among recurrent/salvage arms that explicitly reported non-enhancing/FLAIR-dominant progression and provided an extractable denominator, the exploratory pooled estimate was 27.8% (95% CI 12.8–50.3; k = 6; *n* = 216; I²=75.7%; 95% PI, 3.4–80.6) (Fig. 5).

## Discussion

This systematic review describes how spatial progression is reported across heterogeneous GBM cohorts rather than defining stable recurrence probabilities. First progression after upfront RT/TMZ ± TTF most often included local/in-field enhancing involvement, while progression after a defined salvage line also remained local/in-field in the majority of evaluable cases. At the same time, marginal/field-edge, distant/out-of-field, diffuse/nonlocal, and non-enhancing/FLAIR-dominant events were incompletely and inconsistently reported. High I² values and wide prediction intervals across core endpoints indicate that pooled estimates of reported involvement among evaluable cases should be viewed as descriptive central tendencies across variable studies, not as generalizable patient-level probabilities.

The predominance of local enhancing involvement after upfront therapy aligns with long-standing clinical experience that many first failures occur within or immediately adjacent to previously treated high-risk regions [21, 125, 126]. Although local/in-field involvement was numerically lower after salvage than after upfront therapy, it remained the majority reported pattern among evaluable post-salvage cases. This difference should be interpreted cautiously because later-line cohorts are shaped by survival to salvage, treatment selection, imaging cadence, and ascertainment of patients who remain evaluable [11, 27, 127, 128].

Local, marginal, and distant labels must be interpreted relative to each study’s radiotherapy plan, imaging reference, and operational definition [9–11]. Target-volume practice has evolved over the past two decades, and spatial failure classification differs when studies use single-phase EORTC-style volumes versus two-phase RTOG/NRG-style approaches, when FLAIR abnormality is included or excluded, and when recurrence is classified by isodose line, distance from cavity, or anatomic contiguity [9, 15, 24, 53]. Our harmonization therefore mapped descriptors to local/in-field, marginal/field-edge, and distant/out-of-field involvement within the source study’s own framework rather than treating these labels as geometrically identical across studies.

Non-enhancing/FLAIR-dominant progression was infrequently enumerated, but among studies that explicitly reported it, pooled estimates suggest a meaningful minority of events. This observation is directionally important because it indicates that enhancement-only endpoints may under-ascertain clinically relevant failure, particularly in settings where therapy alters enhancement patterns [10, 129]; however, sparse reporting, heterogeneous definitions, and inconsistent handling of mutual exclusivity (non-enhancing-only vs. mixed) preclude strong inference. Even after overlap adjudication, residual heterogeneity suggests that important drivers were not fully captured by the routinely extractable workbook variables, including extent of resection, baseline multifocality, molecular features, salvage sequencing, and the exact operational definition frameworks used for dose-based, distance-based, and anatomic recurrence classification.

This synthesis is limited by the quality and granularity of the published source data. Individual MR images, radiation plans, pathology slides, molecular reports, and clinical source records were not available; therefore, central pathology or radiology re-review could not be performed. Diagnoses, molecular classifications when reported, progression calls, and spatial categories were accepted as reported by the original investigators. This may introduce diagnostic-era and source-data heterogeneity, particularly in older studies, mixed high-grade glioma or malignant glioma cohorts meeting the prespecified eligibility criteria, and reports using historical GBM terminology. Some older cohorts classified as GBM may include tumors that would now be classified as IDH-mutant grade 4 astrocytoma or, less commonly, other adult diffuse glioma entities under contemporary WHO criteria [14, 108, 130].

Radiographic progression assessment also varied across eras and studies because of differences in imaging cadence, response criteria, steroid or antiangiogenic treatment context, and definitions of local, marginal, distant, diffuse, and non-enhancing/FLAIR-dominant progression. This is especially relevant for bevacizumab-treated cohorts, in which contrast enhancement may be dissociated from tumor burden. Because progression was defined from reported imaging assessments, this synthesis also could not account for radiographically non-evident or microscopic tumor progression not apparent on conventional MRI [131].

Posterior fossa, brainstem, leptomeningeal, and other discontiguous progression patterns deserve separate enumeration because they may be clinically important but are typically collapsed into broad distant or disseminated categories [131–134]. Future recurrence-pattern studies should report these patterns separately from supratentorial parenchymal distant progression, particularly in adult diffuse glioma cohorts where posterior fossa or brainstem involvement may alter prognosis, symptoms, and salvage-treatment feasibility [131, 132, 134].

Because most studies reported arm-level aggregate data, denominators varied across reports, and location categories were frequently non-mutually exclusive; therefore, pooled estimates should be interpreted as involvement proportions rather than exclusive failure-mode partitions. Salvage cohorts were heterogeneous with respect to treatment line, sequencing, re-irradiation approach, bevacizumab co-administration, and systemic therapy exposure; subgroup comparisons should be interpreted descriptively. Non-enhancing/FLAIR-dominant progression was explicitly enumerated in only a small subset of studies, limiting precision. Finally, included cohorts were drawn from clinical trials and institutional series and may not fully represent the broader adult GBM population.

## Conclusions

Across reported GBM cohorts, local/in-field enhancing involvement was the dominant reported pattern at first progression after upfront RT/TMZ ± TTF (~ 80%). Progression after a defined salvage therapy line also remained local/in-field in the majority of evaluable cases (~ 60%), although marginal/field-edge, distant/out-of-field, diffuse/nonlocal, multifocal/disseminated, and non-enhancing/FLAIR-dominant patterns were variably and incompletely reported. The supplementary broad escape-pattern estimate (~ 39%) should be interpreted only as a descriptive sensitivity summary of progression beyond purely local/in-field enhancing failure, not as a standardized recurrence category.

Given high between-study heterogeneity and source-data limitations, these estimates should not be interpreted as definitive recurrence probabilities for contemporary molecular-era IDH-wildtype glioblastoma. Source studies generally lacked central pathology review, central radiology review, uniform molecular classification, standardized spatial definitions, consistent endpoint-specific denominators, complete enumeration of non-enhancing/FLAIR-dominant progression, and uniform follow-up. Future studies should report mutually exclusive failure patterns and non-mutually exclusive involvement categories separately, specify the radiotherapy reference volume/isodose or distance framework, state the evaluable denominator for each endpoint, and separately enumerate posterior fossa/brainstem, leptomeningeal, multifocal/disseminated, and non-enhancing/FLAIR-dominant progression.

## Supplementary Information

Below is the link to the electronic supplementary material.


Supplementary Material 1



Supplementary Material 2


## Data Availability

The data underlying this article were derived from previously published studies. The extracted dataset and analytic code are available from the corresponding author on reasonable request.
