# Supplementary material for: Spatial patterns of progression in reported glioblastoma cohorts after upfront chemoradiation and salvage therapy: a systematic review and meta-analysis
Source: J Neurooncol. 2026 Jul 30;179(1):20. doi: 10.1007/s11060-026-05734-w (PMC13424794; doi:10.1007/s11060-026-05734-w)
Supplement: Supplementary file 2 — Supplementary Material 2 [file 11060_2026_5734_MOESM2_ESM.docx]

**Spatial patterns of progression in reported glioblastoma cohorts after upfront chemoradiation and salvage therapy: a systematic review and meta-analysis**

**Authors:** Rafal Chojak^1,2^, Noah B Drewes^1,2^, Katarzyna Slychan^3^, Rimas V Lukas^2,4^

^1^Department of Neurological Surgery, Feinberg School of Medicine, Northwestern University, Chicago, IL, 60611, USA.

^2^Malnati Brain Tumor Institute of the Robert H. Lurie Comprehensive Cancer Center, Feinberg School of Medicine, Northwestern University, Chicago, IL, 60611, USA.

^3^Department of Neurosurgery, St Lucas Hospital, Tarnow, Poland

^4^Department of Neurology, Northwestern University Feinberg School of Medicine, Chicago, IL, USA.

**Corresponding author:** Rafal Chojak, Department of Neurological Surgery, Feinberg School of Medicine, Northwestern University, Chicago, IL, 60611, USA. ([rafal.chojak@northwestern.edu](mailto:rafal.chojak@northwestern.edu))

**Keywords:** Glioblastoma; Patterns of failure; Recurrence; Chemoradiation; Salvage therapy

**Supplementary data**

**Supplementary table 1.** Search syntax

| **PubMed N = 1221** |
| --- |
| (  "Glioblastoma"[MeSH Terms]  OR glioblastoma*[Title/Abstract]  OR GBM[Title/Abstract]  OR "glioblastoma multiforme"[Title/Abstract]  )  AND  (  "Neoplasm Recurrence, Local"[MeSH Terms]  OR "Neoplasm Recurrence"[MeSH Terms]  OR "Disease Progression"[MeSH Terms]  OR "Treatment Failure"[MeSH Terms]  OR recurren*[Title/Abstract]  OR relapse*[Title/Abstract]  OR reoccur*[Title/Abstract]  OR progress*[Title/Abstract]  OR "treatment failure"[Title/Abstract]  )  AND  (  "pattern of failure"[Title/Abstract]  OR "patterns of failure"[Title/Abstract]  OR "failure pattern"[Title/Abstract]  OR "failure patterns"[Title/Abstract]  OR "pattern of recurrence"[Title/Abstract]  OR "patterns of recurrence"[Title/Abstract]  OR "recurrence pattern"[Title/Abstract]  OR "recurrence patterns"[Title/Abstract]  OR "site of failure"[Title/Abstract]  OR "sites of failure"[Title/Abstract]  OR "site of recurrence"[Title/Abstract]  OR "sites of recurrence"[Title/Abstract]  OR "location of recurrence"[Title/Abstract]  OR topograph*[Title/Abstract]  OR spatial[Title/Abstract]  OR "in-field"[Title/Abstract] OR "in field"[Title/Abstract] OR infield[Title/Abstract]  OR "out-of-field"[Title/Abstract] OR "out of field"[Title/Abstract] OR outfield[Title/Abstract]  OR marginal[Title/Abstract]  OR distant[Title/Abstract]  OR remote[Title/Abstract]  OR disseminat*[Title/Abstract]  OR multifocal*[Title/Abstract]  OR isodose[Title/Abstract]  OR "isodose line"[Title/Abstract]  OR PTV[Title/Abstract]  OR CTV[Title/Abstract]  OR GTV[Title/Abstract]  OR "radiation field"[Title/Abstract]  OR "treatment field"[Title/Abstract]  OR dosimetr*[Title/Abstract]  OR nonenhanc*[Title/Abstract]  OR "non-enhancing"[Title/Abstract]  OR FLAIR[Title/Abstract]  OR "T2/FLAIR"[Title/Abstract]  )  AND humans[MeSH Terms] |

| **Scopus N = 2622** |
| --- |
| TITLE-ABS-KEY(  glioblastoma* OR gbm OR "glioblastoma multiforme"  )  AND TITLE-ABS-KEY(  recurren* OR relapse* OR reoccur* OR progress* OR "treatment failure"  OR "disease progression" OR "neoplasm recurrence" OR "local recurrence"  )  AND TITLE-ABS-KEY(  "pattern of failure" OR "patterns of failure" OR "failure pattern" OR "failure patterns"  OR "pattern of recurrence" OR "patterns of recurrence" OR "recurrence pattern" OR "recurrence patterns"  OR "site of failure" OR "sites of failure" OR "site of recurrence" OR "sites of recurrence"  OR "location of recurrence" OR topograph* OR spatial  OR "in-field" OR "in field" OR infield  OR "out-of-field" OR "out of field" OR outfield  OR marginal OR distant OR remote OR disseminat* OR multifocal*  OR isodose OR "isodose line" OR ptv OR ctv OR gtv  OR "radiation field" OR "treatment field" OR dosimetr*  OR nonenhanc* OR "non-enhancing" OR flair OR "t2/flair"  ) |

**Supplementary table 2. Excluded or Merged Overlapping Reports**

| **Excluded or Merged Overlapping Report** | **Retained Final Study/Rows** | **Rationale** |
| --- | --- | --- |
| **Thomas et al. 2018** | Iwamoto et al. 2009 | Smaller MSKCC post-bevacizumab pathology/autopsy/surgical subset; Iwamoto retained as the more comprehensive recurrent GBM post-BEV failure cohort. |
| **Tini et al. 2018** | Minniti et al. 2023 | Same Siena-linked institutional series with possible February-July 2015 boundary overlap; Minniti retained as the more comprehensive cohort. |
| **Tini et al. 2025** | Minniti et al. 2023 | Siena/linked cohort substantially overlapping the 2015-2020 Minniti cohort; Minniti retained to avoid double counting. |
| **Shimoda et al. 2025** | Yamaki et al. 2020 | Later single-center Tohoku cohort partially overlapping the broader Yamaki Tohoku/Yamagata cohort. |
| **Stewart et al. 2022** | Moore-Palhares et al. 2025 | Sunnybrook/Odette/University of Toronto cohort likely overlaps during 2017-2019; Moore-Palhares retained as the more comprehensive prospective imaging cohort. |
| **Choi et al. 2017** | Yoo et al. 2022_A/B/C | Same Yonsei/Severance institutional system with overlapping upfront recurrence window; Yoo retained as the larger, later subgroup-resolved cohort. |

**Supplementary table 3. Data Extraction and Harmonization Rules**

| During data extraction, we captured surveillance imaging cadence and spatial progression numerators/denominators using prespecified harmonization rules. Imaging cadence (imaging_cadence_weeks) was defined as the planned or typical interval between routine surveillance MRIs during the follow-up window in which progression patterns were assessed; we preferentially extracted explicit schedules (e.g., “every 2 months,” “every 3 months,” “q8–12 weeks”) and converted them to weeks (q2 months = 8; q3 months = 12; q6 weeks = 6). When reported as a range (“every 2–3 months” or “every 8–12 weeks”), we recorded 10 weeks. When schedules changed over time (e.g., q2 months then q3 months), we recorded the earliest/intensive phase unless the pattern-of-failure window was explicitly defined later; if not stated, we recorded NR.  Total cohort at risk (n_total_at_risk) represented the treated/eligible cohort size for the analysis arm (patient-level unless explicitly lesion-level): we used the arm’s cohort N (all treated/eligible), used subgroup denominators when analyses were subgroup-restricted (and appended the subgroup to the study_id to prevent double counting), and set n_total_at_risk to the lesion denominator only for explicitly lesion-level reporting. We did not substitute “number with recurrence” unless the study defined the cohort as recurrent cases only.  For non-enhancing/FLAIR-dominant progression, we extracted n_ne_events only when authors explicitly enumerated NE/FLAIR-dominant events as a distinct endpoint; qualitative mentions, implicit inclusion within spatial categories, or non-countable reporting were coded as NR. We also recorded whether NE events were mutually exclusive from enhancing events (ne_mutually_exclusive) and, when a numeric NE denominator was explicitly stated, entered that denominator in denominator_for_ne. In the synchronized final workbook, denominator_for_ne is stored as the numeric analysis denominator rather than as a separate categorical label. A free-text NE definition was not retained as a standalone workbook column in the final synchronized dataset.  For enhancing spatial outcomes, n_at_risk_enhancing was defined as the number of patients analyzed who were considered at risk for developing an enhancing lesion and were evaluable for spatial classification, according to each study’s reported analysis set.  Enhancing locations were harmonized as involvement counts rather than mutually exclusive failure modes. n_local_involv_enhancing captured in-field/central/local involvement (local-only plus “both local+distant” when provided; otherwise the reported local component in overlapping schemes). n_marginal_enhancing captured field-edge/intermediate involvement (regional/partial-overlap or prespecified distance/isodose zones) and was coded NR when unreported. n_distant_enhancing captured distant/out-of-field involvement per the study’s definition (distant-only plus “both” when provided), without automatically collapsing dissemination subtypes (e.g., subependymal or leptomeningeal spread) into distant unless explicitly included by the authors without separate reporting.  n_diffuse_enhancing captured diffuse/infiltrative enhancing progression only when explicitly enumerated as a separate category; otherwise it was recorded as NR.  Non-local involvement (n_nonlocal_involv_enhancing) was extracted only when the union of marginal and/or distant involvement was explicitly reported (e.g., “non-local/outside field”) or uniquely derivable from mutually exclusive buckets. When studies reported overlapping components, we did not compute unions unless derivable, and dissemination subtypes were included in non-local only when authors defined them within non-local/disseminated categories.  Finally, multifocal_total was extracted only when multifocality was explicitly enumerated (e.g., “multifocal/disseminated”); otherwise it was recorded as NR. |
| --- |

**Supplementary Table 4. Mapping of source-study spatial definitions to harmonized involvement categories.**

| **Source-study descriptor** | **Typical original definition** | **Harmonized category** | **Notes/difficult classifications** |
| --- | --- | --- | --- |
| **In-field / central / local / within high-dose region** | Recurrence or enhancing progression described as local, central, in-field, within the high-dose isodose region, within the treatment volume, or within/overlapping the original tumor bed, GTV, CTV, or PTV. | **Local/in-field involvement** | Mapped using the source study's own dose-volume, distance, or anatomic reference framework. If a source reported both local and distant components, the local component was retained as local involvement. |
| **Local bed / cavity-contact / contiguous recurrence** | Progression contacting or contiguous with the resection cavity, original enhancing lesion, or prior tumor bed when no radiotherapy-plan boundary was provided. | **Local/in-field involvement** | Used for anatomic or descriptor-based studies. Contiguity alone was not used to infer marginal or distant involvement unless the source study separately reported those categories. |
| **Adjacent / marginal / partial overlap / field edge** | Recurrence described as marginal, adjacent to the high-dose region, at the field edge, partially overlapping the high-dose volume, or falling between prespecified isodose or distance thresholds. | **Marginal/field-edge involvement** | Extracted only when the source study separated a marginal or edge category. When the study combined marginal with distant or nonlocal disease and no decomposition was possible, the value was not forced into the marginal endpoint. |
| **Distance-based near-field or borderline recurrence** | Failure categorized by distance from the cavity, enhancing tumor, GTV/CTV/PTV, or prior radiation field, such as within versus beyond a prespecified centimeter threshold. | **Local/in-field, marginal/field-edge, or distant/out-of-field involvement, according to the source threshold** | No patient-level remeasurement was performed. The harmonized category followed the source study's threshold and terminology. |
| **Out-of-field / remote / distant / new noncontiguous lesion** | Progression described as distant, remote, out-of-field, outside the high-dose region or treatment volume, outside the CTV/PTV or specified isodose line, or as a new noncontiguous enhancing lesion. | **Distant/out-of-field involvement** | Includes traditional distant failure descriptors. If a source reported local plus distant disease, the distant component was counted here and the local component was counted in local/in-field involvement. |
| **Local + distant / both** | Simultaneous local/in-field and distant/out-of-field progression in the same patient or arm, including source categories labeled both, mixed local+distant, or local with remote component. | **Counted in both local/in-field and distant/out-of-field involvement** | These are involvement endpoints rather than mutually exclusive pattern bins. Component counts should not be summed across harmonized categories. |
| **Diffuse / nonlocal / infiltrative enhancing pattern** | Diffuse, spreading, invasive, gliomatosis-like, or nonlocal enhancing progression when reported as a spatial pattern rather than a discrete local, marginal, or distant component. | **Extracted separately when reported; included in broad escape only when defined by authors or uniquely derivable** | Difficult because some studies used diffuse to describe morphology, while others used it as a geographic nonlocal category. It was not automatically mapped to distant/out-of-field involvement unless the source definition supported that mapping. |
| **Multifocal/disseminated** | Multiple enhancing foci, multifocal progression, disseminated intracranial disease, leptomeningeal spread, or other non-single-site spread descriptors. | **Extracted separately when reported; included in broad escape only when defined by authors or uniquely derivable** | Mapped conservatively because multifocal disease may include local, marginal, and/or distant components. A multifocal total was not decomposed into specific involvement categories without source-study support. |
| **Non-enhancing/FLAIR-dominant** | Predominantly non-enhancing progression, FLAIR/T2-dominant progression, diffuse non-enhancing progression, or nonenhancing-only progression, particularly in bevacizumab-era or salvage cohorts. | **Non-enhancing/FLAIR-dominant progression** | Extracted only when explicitly enumerated with a clear numerator and denominator. Non-enhancing progression was not inferred from bevacizumab exposure, edema, FLAIR abnormality, or vague diffuse terminology alone. |
| **Enhancing versus non-enhancing source partitions** | Source tables that separated enhancing local/marginal/distant progression from non-enhancing-only or predominantly non-enhancing progression. | **Enhancing involvement endpoints plus separate non-enhancing/FLAIR-dominant endpoint when extractable** | The denominator followed the endpoint-specific evaluable set in the source study. Non-enhancing-only cases were not added to enhancing local, marginal, or distant numerators unless explicitly reported as overlapping. |
| **Composite nonlocal / outside-local descriptors** | Source-study categories such as nonlocal, outside local field, escape, non-central, or beyond local recurrence that combined marginal, distant, diffuse, or multifocal events. | **Broad escape-pattern progression, when author-defined or uniquely derivable** | Used as a supplementary sensitivity construct. If the composite could not be decomposed, it contributed only to broad escape and not to individual marginal or distant endpoints. |
| **Ambiguous, unclear, or framework-mismatch descriptors** | Spatial labels without a usable definition, unclear denominator, incompatible framework, or insufficient information to distinguish local, marginal, distant, diffuse, multifocal, or non-enhancing events. | **Not assigned to a specific harmonized endpoint unless numerically and definitionally extractable** | These data were left blank for the affected endpoint rather than imputed or reclassified. |

**Supplementary table 5. Study-specific mapping of spatial definitions and extracted involvement endpoints.**

| **Source study/arm** | **Year; country; design** | **Analysis context** | **Source spatial framework** | **Extracted endpoints** | **Harmonized categories contributed** | **Mutual exclusivity handling** | **Notes/difficult classifications** |
| --- | --- | --- | --- | --- | --- | --- | --- |
| **Ekinci et al. 2003** | 2003; Turkey; Retrospective | Analysis stratum: Upfront first progression; arm descriptor: None/upfront | Anatomic/descriptor based; Other/unclear; progression: Macdonald | Local: 7/8; Marginal: NR; Distant: 1/8; Broad escape: 1/8; Non-enhancing/FLAIR: NR | Local/in-field; Distant/out-of-field; Broad escape | Harmonized as involvement endpoints; categories may overlap and should not be summed. | In the GBM subgroup (n=25), follow-up MRI showed 7 local regrowth tumors, 1 distant recurrence, and 1 case with no tumor after residual enhancement on early postoperative MRI; the sheet captures the 8 tumor events correctly. \| Escape: Sensitivity column copied from n_nonlocal_involv_enhancing for rows already reporting a nonlocal component. |
| **Giese et al. 2004_A** | 2004; Germany; Clinical trial | Analysis stratum: Upfront first progression; arm descriptor: None/upfront | Anatomic/descriptor based; Anatomic pattern; progression: Macdonald | Local: 11/11; Marginal: NR; Distant: NR; Broad escape: 3/11; Non-enhancing/FLAIR: NR | Local/in-field; Broad escape | Harmonized as involvement endpoints; categories may overlap and should not be summed. | In the GLIADEL arm, local progression was found in 8 of 11 patients and non-local failure in 3; because no failure occurred exclusively away from the original site, the sheet correctly encodes local involvement 11 and nonlocal 3. \| Escape: Sensitivity column copied from n_nonlocal_involv_enhancing for rows already reporting a nonlocal component. |
| **Giese et al. 2004_B** | 2004; Germany; Clinical trial | Analysis stratum: Upfront first progression; arm descriptor: None/upfront | Anatomic/descriptor based; Anatomic pattern; progression: Macdonald | Local: 13/13; Marginal: NR; Distant: NR; Broad escape: 4/13; Non-enhancing/FLAIR: NR | Local/in-field; Broad escape | Harmonized as involvement endpoints; categories may overlap and should not be summed. | In the placebo arm, local progression was found in 9 of 13 patients and non-local failure in 4; because no failure occurred exclusively away from the original site, the sheet correctly encodes local involvement 13 and nonlocal 4. \| Escape: Sensitivity column copied from n_nonlocal_involv_enhancing for rows already reporting a nonlocal component. |
| **Puchner et al. 2004** | 2004; Germany; Clinical trial | Analysis stratum: Upfront first progression; arm descriptor: None/upfront | Anatomic/descriptor based; Anatomic pattern; progression: Macdonald | Local: 33/49; Marginal: NR; Distant: NR; Broad escape: 16/49; Non-enhancing/FLAIR: NR | Local/in-field; Broad escape | Harmonized as involvement endpoints; categories may overlap and should not be summed. | Of 49 evaluable patients after one early non-tumor death, 33 developed primary-site recurrence and 16 developed multifocal tumor recurrence; the sheet matches these local and multifocal totals. \| Escape: Sensitivity-only composite: 16 multifocal recurrences versus 33 primary-site recurrences among 49 evaluable patients. |
| **Souhami et al. 2004_A** | 2004; Multinational; Clinical trial | Analysis stratum: Upfront first progression; arm descriptor: None/upfront | Unclear/NR; Dose-based failure; progression: Macdonald | Local: 72/76; Marginal: 23/76; Distant: 5/76; Broad escape: 25/76; Non-enhancing/FLAIR: NR | Local/in-field; Marginal/field-edge; Distant/out-of-field; Broad escape | Harmonized as involvement endpoints; categories may overlap and should not be summed. | Arm 1 failure categories were local only 51, adjacent only 4, local+adjacent 16, nonadjacent only 0, local+nonadjacent 2, and local+adjacent+nonadjacent 3; the sheet's local 72, marginal 23, distant 5, and nonlocal 25 preserve overlap correctly. \| Escape: Sensitivity column copied from n_nonlocal_involv_enhancing for rows already reporting a nonlocal component. |
| **Souhami et al. 2004_B** | 2004; Multinational; Clinical trial | Analysis stratum: Upfront first progression; arm descriptor: None/upfront | Unclear/NR; Dose-based failure; progression: Macdonald | Local: 66/69; Marginal: 25/69; Distant: 7/69; Broad escape: 27/69; Non-enhancing/FLAIR: NR | Local/in-field; Marginal/field-edge; Distant/out-of-field; Broad escape | Harmonized as involvement endpoints; categories may overlap and should not be summed. | Arm 2 failure categories were local only 42, adjacent only 2, local+adjacent 18, nonadjacent only 1, local+nonadjacent 1, and local+adjacent+nonadjacent 5; the sheet's local 66, marginal 25, distant 7, and nonlocal 27 preserve overlap correctly. \| Escape: Sensitivity column copied from n_nonlocal_involv_enhancing for rows already reporting a nonlocal component. |
| **Chang et al. 2007** | 2007; United States; Retrospective | Analysis stratum: Upfront first progression; arm descriptor: None/upfront | Dose/volume based; Dose-based failure; progression: Macdonald | Local: 43/48; Marginal: 3/48; Distant: 2/48; Broad escape: 5/48; Non-enhancing/FLAIR: NR | Local/in-field; Marginal/field-edge; Distant/out-of-field; Broad escape | Harmonized as involvement endpoints; categories may overlap and should not be summed. | Pattern of failure was 40 central, 3 in-field, 3 marginal, and 2 distant recurrences; the sheet correctly combines central+in-field as 43 local involvement. \| Escape: Sensitivity column copied from n_nonlocal_involv_enhancing for rows already reporting a nonlocal component. |
| **Park et al. 2007** | 2007; United States; Retrospective | Analysis stratum: Upfront first progression; arm descriptor: None/upfront | Dose/volume based; Dose-based failure; progression: NR | Local: 6/9; Marginal: 3/9; Distant: 0/9; Broad escape: 3/9; Non-enhancing/FLAIR: NR | Local/in-field; Marginal/field-edge; Distant/out-of-field; Broad escape | Harmonized as involvement endpoints; categories may overlap and should not be summed. | The pattern table lists 6 contrast-enhancing recurrences entirely covered by 60 Gy and 3 contrast-enhancing recurrences within CNI2 not entirely covered by 60 Gy, with no distant failures; the sheet's local 6, marginal/nonlocal 3, and distant 0 match that classification. \| Escape: Sensitivity column copied from n_nonlocal_involv_enhancing for rows already reporting a nonlocal component. |
| **Showalter et al. 2007** | 2007; United States; Retrospective | Analysis stratum: Upfront first progression; arm descriptor: None/upfront | Anatomic/descriptor based; Anatomic pattern; progression: NR | Local: 49/49; Marginal: NR; Distant: 0/49; Broad escape: 0/49; Non-enhancing/FLAIR: NR | Local/in-field; Distant/out-of-field; Broad escape | Harmonized as involvement endpoints; categories may overlap and should not be summed. | All 49 evaluable patients had local progression with no isolated distant failures. \| Escape: Sensitivity column copied from n_nonlocal_involv_enhancing for rows already reporting a nonlocal component. |
| **Brandes et al. 2009** | 2009; Italy; Prospective | Analysis stratum: Upfront first progression; arm descriptor: None/upfront | Anatomic/descriptor based; Dose-based failure; progression: Macdonald | Local: 57/79; Marginal: 5/79; Distant: 17/79; Broad escape: 22/79; Non-enhancing/FLAIR: NR | Local/in-field; Marginal/field-edge; Distant/out-of-field; Broad escape | Harmonized as involvement endpoints; categories may overlap and should not be summed. | Among 79 recurrences after upfront chemoradiation plus adjuvant TMZ, 57 were in-field, 5 marginal, and 17 outside the radiation field; the sheet preserves local 57 and nonlocal 22 with the reported marginal and distant components. \| Escape: Sensitivity column copied from n_nonlocal_involv_enhancing for rows already reporting a nonlocal component. |
| **Panet-Raymond et al. 2009** | 2009; Canada; Retrospective | Analysis stratum: Upfront first progression; arm descriptor: None/upfront | Distance based; Dose-based failure; progression: NR | Local: 21/23; Marginal: NR; Distant: 2/23; Broad escape: 2/23; Non-enhancing/FLAIR: NR | Local/in-field; Distant/out-of-field; Broad escape | Harmonized as involvement endpoints; categories may overlap and should not be summed. | Final source audit confirmed source text: 23 documented MRI progressions; 21 central and 2 failures >2 cm from the initial GTV. No numerator/denominator change. \| Escape: Sensitivity column copied from n_nonlocal_involv_enhancing for rows already reporting a nonlocal component. |
| **Tuettenberg et al. 2009** | 2009; Germany; Prospective | Analysis stratum: Upfront first progression; arm descriptor: None/upfront | Anatomic/descriptor based; Local/distant pattern; progression: NR | Local: 12/32; Marginal: NR; Distant: 20/32; Broad escape: 20/32; Non-enhancing/FLAIR: NR | Local/in-field; Distant/out-of-field; Broad escape | Harmonized as involvement endpoints; categories may overlap and should not be summed. | Under antiangiogenic therapy, 62.5% of the 32 patients had distant recurrence; because 40% of those occurred with good local control, the sheet appropriately preserves overlap as local 12 and distant/nonlocal 20. \| Escape: Sensitivity column copied from n_nonlocal_involv_enhancing for rows already reporting a nonlocal component. |
| **Milano et al. 2010** | 2010; United States; Retrospective | Analysis stratum: Upfront first progression; arm descriptor: None/upfront | Dose/volume based; Dose-based failure; progression: Macdonald | Local: 36/39; Marginal: 6/39; Distant: 5/39; Broad escape: 11/39; Non-enhancing/FLAIR: NR | Local/in-field; Marginal/field-edge; Distant/out-of-field; Broad escape | Harmonized as involvement endpoints; categories may overlap and should not be summed. | Among 39 recurrent patients, first recurrence included 36 in-field, 6 marginal, and 5 distant lesions; the sheet's local and nonlocal involvement counts are consistent. \| Escape: Sensitivity column copied from n_nonlocal_involv_enhancing for rows already reporting a nonlocal component. |
| **Minniti et al. 2010** | 2010; Italy; Retrospective | Analysis stratum: Upfront first progression; arm descriptor: None/upfront | Dose/volume based; Dose-based failure; progression: Macdonald | Local: 85/105; Marginal: 6/105; Distant: 14/105; Broad escape: 20/105; Non-enhancing/FLAIR: NR | Local/in-field; Marginal/field-edge; Distant/out-of-field; Broad escape | Harmonized as involvement endpoints; categories may overlap and should not be summed. | Recurrences were central in 79, in-field in 6, marginal in 6, and distant in 14 of 105 patients; the sheet correctly combines central+in-field as 85 local involvement. \| Escape: Sensitivity column copied from n_nonlocal_involv_enhancing for rows already reporting a nonlocal component. |
| **Chamberlain et al. 2011_A** | 2011; United States; Retrospective | Analysis stratum: Upfront first progression; arm descriptor: None/upfront | Anatomic/descriptor based; Enhancing vs non-enhancing; progression: Macdonald | Local: 64/80; Marginal: NR; Distant: 6/80; Broad escape: 16/80; Non-enhancing/FLAIR: NR | Local/in-field; Distant/out-of-field; Broad escape | Harmonized as involvement endpoints; categories may overlap and should not be summed. | source confirmed after pdf remap \| Corrected PDF to Chamberlain case report; first recurrence table confirms 64 local, 6 distant, 5 multifocal, 5 diffuse. \| Escape: Sensitivity-only composite: first recurrence table gives 5 diffuse + 6 distant + 5 multifocal mutually exclusive escape-pattern events. \| Overlap: Same 80-patient University of Washington cohort contributes a first-progression row and a later post-BEV row in the same paper; do not treat these rows as independent in any pooled model spanning stages. |
| **Dobelbower et al. 2011** | 2011; United States; Retrospective | Analysis stratum: Upfront first progression; arm descriptor: None/upfront | Dose/volume based; Dose-based failure; progression: Macdonald | Local: 18/20; Marginal: 2/20; Distant: 4/20; Broad escape: 6/20; Non-enhancing/FLAIR: NR | Local/in-field; Marginal/field-edge; Distant/out-of-field; Broad escape | Harmonized as involvement endpoints; categories may overlap and should not be summed. | All 20 failures had a primary-site component; 18 were in-field, 2 marginal, and 4 had an additional distant component. \| Escape: Sensitivity column copied from n_nonlocal_involv_enhancing for rows already reporting a nonlocal component. |
| **McDonald et al. 2011** | 2011; United States; Retrospective | Analysis stratum: Upfront first progression; arm descriptor: None/upfront | Dose/volume based; Dose-based failure; progression: Macdonald | Local: 38/41; Marginal: 2/41; Distant: 1/41; Broad escape: 3/41; Non-enhancing/FLAIR: NR | Local/in-field; Marginal/field-edge; Distant/out-of-field; Broad escape | Harmonized as involvement endpoints; categories may overlap and should not be summed. | Among 41 evaluable recurrences, 38 were central/in-field, 2 marginal, and 1 distant. \| Escape: Sensitivity column copied from n_nonlocal_involv_enhancing for rows already reporting a nonlocal component. |
| **Oh et al. 2011** | 2011; Canada; Retrospective | Analysis stratum: Upfront first progression; arm descriptor: None/upfront | Distance based; Anatomic pattern; progression: RECIST/other | Local: 58/67; Marginal: NR; Distant: 9/67; Broad escape: 9/67; Non-enhancing/FLAIR: NR | Local/in-field; Distant/out-of-field; Broad escape | Harmonized as involvement endpoints; categories may overlap and should not be summed. | First recurrence was local in 58 and distant in 9 of 67 patients. \| Escape: Sensitivity column copied from n_nonlocal_involv_enhancing for rows already reporting a nonlocal component. |
| **Gunjur et al. 2012** | 2012; Australia; Retrospective | Analysis stratum: Upfront first progression; arm descriptor: None/upfront | Unclear/NR; Dose-based failure; progression: Macdonald | Local: 43/47; Marginal: 3/47; Distant: 1/47; Broad escape: 4/47; Non-enhancing/FLAIR: NR | Local/in-field; Marginal/field-edge; Distant/out-of-field; Broad escape | Harmonized as involvement endpoints; categories may overlap and should not be summed. | Among 47 evaluable progressions, 36 were central, 7 in-field non-central, 3 marginal, and 1 out-of-field; the sheet's 43 local and 4 nonlocal counts are correct. \| Escape: Sensitivity column copied from n_nonlocal_involv_enhancing for rows already reporting a nonlocal component. |
| **Konishi et al. 2012** | 2012; Japan; Retrospective | Analysis stratum: Upfront first progression; arm descriptor: None/upfront | Distance based; Dose-based failure; progression: RANO or Macdonald | Local: 20/33; Marginal: 2/33; Distant: 4/33; Broad escape: 10/33; Non-enhancing/FLAIR: NR | Local/in-field; Marginal/field-edge; Distant/out-of-field; Broad escape | Harmonized as involvement endpoints; categories may overlap and should not be summed. | Among 33 recurrences, 20 were regional, 2 marginal, 4 distant, 4 multiple, and 3 subarachnoid dissemination; the sheet matches the extracted regional/marginal/distant/multiple fields and leaves dissemination outside existing columns. \| Escape: Sensitivity-only composite: mutually exclusive recurrence categories give 2 marginal + 4 distant + 4 multiple recurrences; subarachnoid dissemination was excluded. |
| **Monjazeb et al. 2012** | 2012; United States; Prospective | Analysis stratum: Upfront first progression; arm descriptor: None/upfront | Dose/volume based; Dose-based failure; progression: NR | Local: 17/18; Marginal: 0/18; Distant: 1/18; Broad escape: 1/18; Non-enhancing/FLAIR: NR | Local/in-field; Marginal/field-edge; Distant/out-of-field; Broad escape | Harmonized as involvement endpoints; categories may overlap and should not be summed. | Of 21 enrolled patients, first progression was in-field in 17 and out-of-field in 1, while 3 never had radiographic or pathologic progression; the sheet matches this 18-event denominator. \| Escape: Sensitivity column copied from n_nonlocal_involv_enhancing for rows already reporting a nonlocal component. |
| **Niyazi et al. 2012** | 2012; Germany; Retrospective | Analysis stratum: Upfront first progression; arm descriptor: None/upfront | Dose/volume based; Dose-based failure; progression: Macdonald | Local: 39/52; Marginal: 3/52; Distant: 10/52; Broad escape: 13/52; Non-enhancing/FLAIR: NR | Local/in-field; Marginal/field-edge; Distant/out-of-field; Broad escape | Harmonized as involvement endpoints; categories may overlap and should not be summed. | Of 79 patients, 39 had in-field recurrence, 3 marginal recurrence, 10 ex-field recurrence, and 27 no relapse; the sheet matches the 52 progressing patients. \| Escape: Sensitivity column copied from n_nonlocal_involv_enhancing for rows already reporting a nonlocal component. |
| **Piroth et al. 2012** | 2012; Germany; Prospective | Analysis stratum: Upfront first progression; arm descriptor: None/upfront | Dose/volume based; Dose-based failure; progression: Macdonald | Local: 17/18; Marginal: NR; Distant: 4/18; Broad escape: 4/18; Non-enhancing/FLAIR: NR | Local/in-field; Distant/out-of-field; Broad escape | Harmonized as involvement endpoints; categories may overlap and should not be summed. | Overall, 14 local, 3 local+distant, and 1 distant-only relapses were recorded; the sheet correctly encodes local 17 and distant/nonlocal 4. \| Escape: Sensitivity column copied from n_nonlocal_involv_enhancing for rows already reporting a nonlocal component. |
| **De Bonis et al. 2013** | 2013; Italy; Retrospective | Analysis stratum: Upfront first progression; arm descriptor: None/upfront | Distance based; Dose-based failure; progression: Macdonald | Local: 60/75; Marginal: NR; Distant: 15/75; Broad escape: 15/75; Non-enhancing/FLAIR: NR | Local/in-field; Distant/out-of-field; Broad escape | Harmonized as involvement endpoints; categories may overlap and should not be summed. | Among the 88 gross-total-resection patients, recurrence occurred in 75 cases: 60 local and 15 distant; the sheet matches these totals. \| Escape: Sensitivity column copied from n_nonlocal_involv_enhancing for rows already reporting a nonlocal component. |
| **Dorner et al. 2013** | 2013; Germany; Prospective | Analysis stratum: Upfront first progression; arm descriptor: None/upfront | Anatomic/descriptor based; Anatomic pattern; progression: NR | Local: 13/19; Marginal: NR; Distant: 5/19; Broad escape: 5/19; Non-enhancing/FLAIR: NR | Local/in-field; Distant/out-of-field; Broad escape | Harmonized as involvement endpoints; categories may overlap and should not be summed. | Primary-glioma subgroup (n=19) had 8 local, 6 diffuse, and 5 multilocular recurrences; the sheet correctly encodes local involvement as 13 and nonlocal involvement as 5. \| Escape: Sensitivity column copied from n_nonlocal_involv_enhancing for rows already reporting a nonlocal component. |
| **Ho et al. 2013** | 2013; United States; Retrospective | Analysis stratum: Upfront first progression; arm descriptor: None/upfront | Unclear/NR; Dose-based failure; progression: RANO or Macdonald | Local: 48/56; Marginal: 4/56; Distant: 3/56; Broad escape: 7/56; Non-enhancing/FLAIR: NR | Local/in-field; Marginal/field-edge; Distant/out-of-field; Broad escape | Harmonized as involvement endpoints; categories may overlap and should not be summed. | Among 56 MRI-documented recurrences, 48 were central, 4 marginal, and 3 distant; the sheet's local, marginal, and distant counts match the source. \| Escape: Sensitivity column copied from n_nonlocal_involv_enhancing for rows already reporting a nonlocal component. |
| **Kimura et al. 2013** | 2013; United States; Retrospective | Analysis stratum: Upfront first progression; arm descriptor: None/upfront | Anatomic/descriptor based; Anatomic contact pattern; progression: NR | Local: 21/49; Marginal: 22/49; Distant: 6/49; Broad escape: 28/49; Non-enhancing/FLAIR: NR | Local/in-field; Marginal/field-edge; Distant/out-of-field; Broad escape | Harmonized as involvement endpoints; categories may overlap and should not be summed. | Overall recurrence patterns were 21 local, 22 spread, and 6 distant among 49 cases; the sheet's local, intermediate, and distant counts match the source. \| Escape: Sensitivity column copied from n_nonlocal_involv_enhancing for rows already reporting a nonlocal component. |
| **Ogura et al. 2013** | 2013; Japan; Retrospective | Analysis stratum: Upfront first progression; arm descriptor: None/upfront | Dose/volume based; Dose-based failure; progression: RANO or Macdonald | Local: 17/21; Marginal: 0/21; Distant: 4/21; Broad escape: 6/21; Non-enhancing/FLAIR: NR | Local/in-field; Marginal/field-edge; Distant/out-of-field; Broad escape | Harmonized as involvement endpoints; categories may overlap and should not be summed. | Initial recurrences were central in 14, in-field in 4, out-field in 2, and distant in 4, with one central+in-field and two central+distant overlaps; the sheet's involvement counts are consistent. \| Escape: Sensitivity column copied from n_nonlocal_involv_enhancing for rows already reporting a nonlocal component. |
| **Petrecca et al. 2013** | 2013; Canada; Retrospective | Analysis stratum: Upfront first progression; arm descriptor: None/upfront | Anatomic/descriptor based; Dose-based failure; progression: RANO | Local: 18/20; Marginal: NR; Distant: 3/20; Broad escape: 3/20; Non-enhancing/FLAIR: NR | Local/in-field; Distant/out-of-field; Broad escape | Harmonized as involvement endpoints; categories may overlap and should not be summed. | Recurrence was restricted to the resection margin in 17 cases, distant only in 2, and both local+distant in 1; the sheet correctly encodes local 18 and nonlocal/distant 3. \| Escape: Sensitivity column copied from n_nonlocal_involv_enhancing for rows already reporting a nonlocal component. |
| **Sherriff et al. 2013** | 2013; United Kingdom; Retrospective | Analysis stratum: Upfront first progression; arm descriptor: None/upfront | Dose/volume based; Dose-based failure; progression: Macdonald | Local: 55/71; Marginal: 0/71; Distant: 16/71; Broad escape: 16/71; Non-enhancing/FLAIR: NR | Local/in-field; Marginal/field-edge; Distant/out-of-field; Broad escape | Harmonized as involvement endpoints; categories may overlap and should not be summed. | Among 71 MRI-confirmed intracranial relapses, 55 were central after plan review and 16 were distant; marginal candidates were reclassified as in-field/central. \| Escape: Sensitivity column copied from n_nonlocal_involv_enhancing for rows already reporting a nonlocal component. |
| **Shields et al. 2013** | 2013; United States; Retrospective | Analysis stratum: Upfront first progression; arm descriptor: None/upfront | Dose/volume based; Dose-based failure; progression: RANO or Macdonald | Local: 3/12; Marginal: 2/12; Distant: 7/12; Broad escape: 9/12; Non-enhancing/FLAIR: NR | Local/in-field; Marginal/field-edge; Distant/out-of-field; Broad escape | Harmonized as involvement endpoints; categories may overlap and should not be summed. | Among 12 recurrences, 3 were in-field, 2 marginal, and 7 distant with subependymal or deep white matter spread; the sheet correctly records local 3 and nonlocal 9. \| Escape: Sensitivity column copied from n_nonlocal_involv_enhancing for rows already reporting a nonlocal component. |
| **Adeberg et al. 2014** | 2014; Germany; Retrospective | Analysis stratum: Upfront first progression; arm descriptor: None/upfront | Anatomic/descriptor based; Dose-based failure; progression: NR | Local: 410/424; Marginal: NR; Distant: NR; Broad escape: 163/424; Non-enhancing/FLAIR: NR | Local/in-field; Broad escape | Harmonized as involvement endpoints; categories may overlap and should not be summed. | Of 424 classifiable progressions, 261 were local, 11 ipsilateral, 2 contralateral, 1 splenial, 75 local+ipsilateral, and 74 local+contralateral; the sheet's local/nonlocal involvement counts are consistent. \| Escape: Sensitivity column copied from n_nonlocal_involv_enhancing for rows already reporting a nonlocal component. |
| **Iuchi et al. 2014** | 2014; Japan; Clinical trial | Analysis stratum: Upfront first progression; arm descriptor: None/upfront | Dose/volume based; Dose-based failure; progression: NR | Local: 11/30; Marginal: NR; Distant: 5/30; Broad escape: 19/30; Non-enhancing/FLAIR: NR | Local/in-field; Distant/out-of-field; Broad escape | Harmonized as involvement endpoints; categories may overlap and should not be summed. | Among 30 primary failures, the primary site of failure was local in 11 patients, distant in 5, and beside the CSF space in 14; the sheet correctly records local 11 and nonlocal 19 with 5 distant failures. \| Escape: Sensitivity column copied from n_nonlocal_involv_enhancing for rows already reporting a nonlocal component. |
| **Miwa et al. 2014** | 2014; Japan; Prospective | Analysis stratum: Upfront first progression; arm descriptor: None/upfront | Dose/volume based; Dose-based failure; progression: NR | Local: 7/28; Marginal: NR; Distant: 4/28; Broad escape: 21/28; Non-enhancing/FLAIR: NR | Local/in-field; Distant/out-of-field; Broad escape | Harmonized as involvement endpoints; categories may overlap and should not be summed. | Among 28 failures after hypo-IMRT, 17 were CSF dissemination, 7 local progression, and 4 distant failure; the sheet correctly records local 7 and nonlocal 21 with 4 distant-parenchymal events. \| Escape: Sensitivity column copied from n_nonlocal_involv_enhancing for rows already reporting a nonlocal component. |
| **Omuro et al. 2014** | 2014; United States; Clinical trial | Analysis stratum: Upfront first progression; arm descriptor: None/upfront | Unclear/NR; Dose-based failure; progression: RANO or Macdonald | Local: 25/29; Marginal: NR; Distant: 4/29; Broad escape: 4/29; Non-enhancing/FLAIR: NR | Local/in-field; Distant/out-of-field; Broad escape | Harmonized as involvement endpoints; categories may overlap and should not be summed. | Radiographic progression pattern was determined in 29 trial patients: 25 local and 4 distant, with no diffuse/multifocal pattern reported. \| Escape: Sensitivity column copied from n_nonlocal_involv_enhancing for rows already reporting a nonlocal component. |
| **Reddy et al. 2014** | 2014; United States; Clinical trial | Analysis stratum: Upfront first progression; arm descriptor: None/upfront | Unclear/NR; Dose-based failure; progression: RANO or Macdonald | Local: 7/17; Marginal: 2/17; Distant: 9/17; Broad escape: 11/17; Non-enhancing/FLAIR: NR | Local/in-field; Marginal/field-edge; Distant/out-of-field; Broad escape | Harmonized as involvement endpoints; categories may overlap and should not be summed. | Seventeen of 24 patients had radiographic failure: 1 central, 5 in-field, 2 marginal, 8 distant, and 1 both in-field and distant; the sheet correctly preserves overlap as local 7 and distant/nonlocal 11. \| Escape: Sensitivity column copied from n_nonlocal_involv_enhancing for rows already reporting a nonlocal component. |
| **Tejada et al. 2014** | 2014; Spain; Retrospective | Analysis stratum: Upfront first progression; arm descriptor: None/upfront | Anatomic/descriptor based; Anatomic contact pattern; progression: RANO or Macdonald | Local: 42/58; Marginal: NR; Distant: 20/58; Broad escape: 20/58; Non-enhancing/FLAIR: NR | Local/in-field; Distant/out-of-field; Broad escape | Harmonized as involvement endpoints; categories may overlap and should not be summed. | Among 58 first recurrences, 38 were local only, 16 distant only, and 4 simultaneous local+distant; the sheet correctly yields local 42 and nonlocal 20. \| Escape: Sensitivity column copied from n_nonlocal_involv_enhancing for rows already reporting a nonlocal component. |
| **Chen et al. 2015** | 2015; United States; Retrospective | Analysis stratum: Upfront first progression; arm descriptor: None/upfront | Dose/volume based; Dose-based failure; progression: NR | Local: 79/102; Marginal: NR; Distant: 23/102; Broad escape: 23/102; Non-enhancing/FLAIR: NR | Local/in-field; Distant/out-of-field; Broad escape | Harmonized as involvement endpoints; categories may overlap and should not be summed. | Source reports 79 local recurrences within field and 23 distant recurrences among 102 tumors. \| Escape: Sensitivity column copied from n_nonlocal_involv_enhancing for rows already reporting a nonlocal component. |
| **Elson et al. 2015** | 2015; United States; Retrospective | Analysis stratum: Upfront first progression; arm descriptor: None/upfront | Dose/volume based; Dose-based failure; progression: NR | Local: 27/32; Marginal: 4/32; Distant: 1/32; Broad escape: 5/32; Non-enhancing/FLAIR: NR | Local/in-field; Marginal/field-edge; Distant/out-of-field; Broad escape | Harmonized as involvement endpoints; categories may overlap and should not be summed. | Among 32 analyzed recurrences, 27 were central, 4 marginal, and 1 distant. \| Escape: Sensitivity column copied from n_nonlocal_involv_enhancing for rows already reporting a nonlocal component. |
| **Nestler et al. 2015** | 2015; Germany; Post hoc | Analysis stratum: Upfront first progression; arm descriptor: None/upfront | Anatomic/descriptor based; Anatomic contact pattern; progression: RECIST/other | Local: 80/83; Marginal: NR; Distant: 10/83; Broad escape: 10/83; Non-enhancing/FLAIR: NR | Local/in-field; Distant/out-of-field; Broad escape | Harmonized as involvement endpoints; categories may overlap and should not be summed. | In 83 recurrences, 80 involved the former resection margin; 3 were distant-only and 7 had simultaneous local+distant recurrence, while multiple nodular recurrences were seen in 40 patients. \| Escape: Sensitivity column copied from n_nonlocal_involv_enhancing for rows already reporting a nonlocal component. |
| **Ney et al. 2015** | 2015; United States; Clinical trial | Analysis stratum: Upfront first progression; arm descriptor: None/upfront | Dose/volume based; Dose-based failure; progression: Macdonald | Local: 20/25; Marginal: NR; Distant: 9/25; Broad escape: 9/25; Non-enhancing/FLAIR: NR | Local/in-field; Distant/out-of-field; Broad escape | Harmonized as involvement endpoints; categories may overlap and should not be summed. | Among 25 evaluable radiographic progressions, 16 were local only, 4 were both local and outside-field, and 5 were outside-field only; the sheet correctly preserves overlap as local 20 and distant/nonlocal 9. \| Escape: Sensitivity column copied from n_nonlocal_involv_enhancing for rows already reporting a nonlocal component. |
| **Thiepold et al. 2015_A** | 2015; Germany; Retrospective | Analysis stratum: Upfront first progression; arm descriptor: None/upfront | Anatomic/descriptor based; Anatomic pattern; progression: NR | Local: 29/36; Marginal: NR; Distant: 5/36; Broad escape: 7/36; Non-enhancing/FLAIR: NR | Local/in-field; Distant/out-of-field; Broad escape | Harmonized as involvement endpoints; categories may overlap and should not be summed. | source confirmed after pdf remap \| Source PDF was remapped to the added Thiepold ischemia paper. The abstract reports diffuse or distant recurrence in 19.4% of controls, matching the sheet's 7 of 36 control-cohort escape events (2 diffuse + 5 distant). \| Escape: Sensitivity-only composite: remapped Thiepold control cohort shows 29 local, 2 diffuse, and 5 distant recurrences among 36 evaluable failures, so escape equals 2 + 5. |
| **Thiepold et al. 2015_B** | 2015; Germany; Retrospective | Analysis stratum: Upfront first progression; arm descriptor: None/upfront | Anatomic/descriptor based; Anatomic pattern; progression: NR | Local: 14/36; Marginal: NR; Distant: 12/36; Broad escape: 22/36; Non-enhancing/FLAIR: NR | Local/in-field; Distant/out-of-field; Broad escape | Harmonized as involvement endpoints; categories may overlap and should not be summed. | source confirmed after pdf remap \| Source PDF was remapped to the added Thiepold ischemia paper. The abstract reports diffuse or distant recurrence in 61.1% of the perioperative-ischemia cohort, matching the sheet's 22 of 36 escape events (10 diffuse + 12 distant). \| Escape: Sensitivity-only composite: remapped Thiepold perioperative-ischemia cohort shows 14 local, 10 diffuse, and 12 distant recurrences among 36 evaluable failures, so escape equals 10 + 12. |
| **Buglione et al. 2016** | 2016; Italy; Retrospective | Analysis stratum: Upfront first progression; arm descriptor: None/upfront | Dose/volume based; Dose-based failure; progression: Macdonald | Local: 60/68; Marginal: 7/68; Distant: 1/68; Broad escape: 8/68; Non-enhancing/FLAIR: NR | Local/in-field; Marginal/field-edge; Distant/out-of-field; Broad escape | Harmonized as involvement endpoints; categories may overlap and should not be summed. | Local recurrence analysis cohort of 68 shows 60 in-field, 7 marginal, and 1 distant failures. \| Escape: Sensitivity column copied from n_nonlocal_involv_enhancing for rows already reporting a nonlocal component. |
| **Harat et al. 2016** | 2016; Poland; Prospective | Analysis stratum: Upfront first progression; arm descriptor: None/upfront | Dose/volume based; Dose-based failure; progression: NR | Local: 13/23; Marginal: 3/23; Distant: 5/23; Broad escape: 8/23; Non-enhancing/FLAIR: NR | Local/in-field; Marginal/field-edge; Distant/out-of-field; Broad escape | Harmonized as involvement endpoints; categories may overlap and should not be summed. | Using the GTVRM framework in Table 3, progression was 57% central, 13% in-field, 21% out-of-field, and 9% multifocal; for 23 events this corresponds to 13, 3, 5, and 2, matching the sheet. \| Escape: Sensitivity column copied from n_nonlocal_involv_enhancing for rows already reporting a nonlocal component. |
| **Wick et al. 2016_A** | 2016; Multinational; Clinical trial | Analysis stratum: Upfront first progression; arm descriptor: None/upfront | Anatomic/descriptor based; Enhancing vs non-enhancing; progression: RANO or Macdonald | Local: 208/299; Marginal: NR; Distant: 15/299; Broad escape: NR; Non-enhancing/FLAIR: 51/354 | Local/in-field; Distant/out-of-field; Non-enhancing/FLAIR | Harmonized as involvement endpoints; categories may overlap and should not be summed. | In the BEV arm, 299 patients had baseline and progression focus data: 198 local->local, 57 multifocal->multifocal, 19 local->multifocal, 11 local->distant, 9 multifocal->local, 4 multifocal->distant, and 1 no-pattern->local; the sheet's local 208, distant 15, and multifocal 76 are correct. \| Escape: Left blank intentionally: progression categories mix local, distant, and multifocal focus-pattern states rather than a harmonizable nonlocal-or-infiltrative escape union on the current denominator framework. |
| **Wick et al. 2016_B** | 2016; Multinational; Clinical trial | Analysis stratum: Upfront first progression; arm descriptor: None/upfront | Anatomic/descriptor based; Enhancing vs non-enhancing; progression: RANO or Macdonald | Local: 217/333; Marginal: NR; Distant: 17/333; Broad escape: NR; Non-enhancing/FLAIR: 53/387 | Local/in-field; Distant/out-of-field; Non-enhancing/FLAIR | Harmonized as involvement endpoints; categories may overlap and should not be summed. | In the placebo arm, 333 patients had baseline and progression focus data: 215 local->local, 63 multifocal->multifocal, 30 local->multifocal, 15 local->distant, 8 multifocal->local, and 2 multifocal->distant; the sheet's local 217, distant 17, and multifocal 93 are correct. \| Escape: Left blank intentionally: progression categories mix local, distant, and multifocal focus-pattern states rather than a harmonizable nonlocal-or-infiltrative escape union on the current denominator framework. |
| **Lundemann et al. 2017** | 2017; Denmark; Retrospective | Analysis stratum: Upfront first progression; arm descriptor: None/upfront | Dose/volume based; Dose-based failure; progression: RANO | Local: 46/50; Marginal: 1/50; Distant: 3/50; Broad escape: 4/50; Non-enhancing/FLAIR: NR | Local/in-field; Marginal/field-edge; Distant/out-of-field; Broad escape | Harmonized as involvement endpoints; categories may overlap and should not be summed. | Final source audit confirmed source text/Table 2: 50 eligible for pattern-of-failure analysis; central 41 plus in-field 5 mapped to local/in-field 46; marginal 1; distant 3. No numerator/denominator change. \| Escape: Sensitivity column copied from n_nonlocal_involv_enhancing for rows already reporting a nonlocal component. |
| **Pessina et al. 2017** | 2017; Italy; Retrospective | Analysis stratum: Upfront first progression; arm descriptor: None/upfront | Unclear/NR; Enhancing vs non-enhancing; progression: NR | Local: 206/220; Marginal: NR; Distant: 32/220; Broad escape: 32/220; Non-enhancing/FLAIR: NR | Local/in-field; Distant/out-of-field; Broad escape | Harmonized as involvement endpoints; categories may overlap and should not be summed. | Among 220 pattern-classified recurrences, 206 occurred at the treated site, including 18 with concurrent distant progression, and 14 were distant only; the sheet correctly encodes local 206 and nonlocal/distant 32. \| Escape: Sensitivity column copied from n_nonlocal_involv_enhancing for rows already reporting a nonlocal component. |
| **Rapp et al. 2017** | 2017; Germany; Retrospective | Analysis stratum: Upfront first progression; arm descriptor: None/upfront | Distance based; Anatomic pattern; progression: RANO or Macdonald | Local: 87/97; Marginal: NR; Distant: 20/97; Broad escape: 20/97; Non-enhancing/FLAIR: NR | Local/in-field; Distant/out-of-field; Broad escape | Harmonized as involvement endpoints; categories may overlap and should not be summed. | Among 97 histologically proven recurrences, 77 were local, 10 distant, and 10 combined local+distant; the sheet correctly preserves overlap as local 87 and nonlocal/distant 20. \| Escape: Sensitivity column copied from n_nonlocal_involv_enhancing for rows already reporting a nonlocal component. |
| **Yan et al. 2017** | 2017; United Kingdom; Retrospective | Analysis stratum: Upfront first progression; arm descriptor: None/upfront | Anatomic/descriptor based; Other/unclear; progression: NR | Local: 28/31; Marginal: NR; Distant: 5/31; Broad escape: 5/31; Non-enhancing/FLAIR: NR | Local/in-field; Distant/out-of-field; Broad escape | Harmonized as involvement endpoints; categories may overlap and should not be summed. | There were 26 local recurrences, 3 distal recurrences, and 2 local+distal recurrences; the sheet's local 28 and distant/nonlocal 5 are correct. \| Escape: Sensitivity column copied from n_nonlocal_involv_enhancing for rows already reporting a nonlocal component. |
| **Bette et al. 2018** | 2018; Germany; Retrospective | Analysis stratum: Upfront first progression; arm descriptor: None/upfront | Anatomic/descriptor based; Anatomic contact pattern; progression: RANO | Local: 116/129; Marginal: NR; Distant: 57/129; Broad escape: 57/129; Non-enhancing/FLAIR: NR | Local/in-field; Distant/out-of-field; Broad escape | Harmonized as involvement endpoints; categories may overlap and should not be summed. | Among 129 first recurrences, 72 were local only, 13 distant only, and 44 local+distant; this yields local 116 and distant/nonlocal 57, with 85 multifocal recurrences. \| Escape: Sensitivity column copied from n_nonlocal_involv_enhancing for rows already reporting a nonlocal component. |
| **Jayamanne et al. 2018** | 2018; Australia; Retrospective | Analysis stratum: Upfront first progression; arm descriptor: None/upfront | Distance based; Dose-based failure; progression: RANO or Macdonald | Local: 72/86; Marginal: 41/86; Distant: 22/86; Broad escape: NR; Non-enhancing/FLAIR: NR | Local/in-field; Marginal/field-edge; Distant/out-of-field | Harmonized as involvement endpoints; categories may overlap and should not be summed. | At initial relapse, 72 patients had a component of local failure, 41 had regional-site involvement, and 22 had distant-site involvement among 86 recurrences; the sheet's local, marginal/regional, and distant counts match the paper. \| Escape: Left blank intentionally: the source reports overlapping local, regional, and distant-site involvement, so a unique escape-pattern union cannot be derived without double counting. |
| **Rowe et al. 2018** | 2018; United States; Retrospective | Analysis stratum: Upfront first progression; arm descriptor: None/upfront | Unclear/NR; Dose-based failure; progression: RANO | Local: 53/55; Marginal: NR; Distant: 2/55; Broad escape: 2/55; Non-enhancing/FLAIR: NR | Local/in-field; Distant/out-of-field; Broad escape | Harmonized as involvement endpoints; categories may overlap and should not be summed. | Among 55 patients with known recurrence, 53 were local within the 90% isodose and 2 were nonlocal. \| Escape: Sensitivity column copied from n_nonlocal_involv_enhancing for rows already reporting a nonlocal component. |
| **Schaub et al. 2018** | 2018; Germany; Post hoc | Analysis stratum: Upfront first progression; arm descriptor: None/upfront | Anatomic/descriptor based; Anatomic pattern; progression: NR | Local: 89/142; Marginal: NR; Distant: 22/142; Broad escape: 53/142; Non-enhancing/FLAIR: NR | Local/in-field; Distant/out-of-field; Broad escape | Harmonized as involvement endpoints; categories may overlap and should not be summed. | At first recurrence, the combined BEV/IRI and TMZ cohorts had 89 local, 31 multifocal, and 22 distant growth patterns among 142 evaluable patients; the sheet matches these totals. \| Escape: Sensitivity-only composite: mutually exclusive first-recurrence growth patterns yield 31 multifocal + 22 distant events among 142 evaluable patients. |
| **Kim et al. 2019** | 2019; United States; Retrospective | Analysis stratum: Upfront first progression; arm descriptor: None/upfront | Dose/volume based; Dose-based failure; progression: NR | Local: 36/68; Marginal: 16/68; Distant: 16/68; Broad escape: 32/68; Non-enhancing/FLAIR: NR | Local/in-field; Marginal/field-edge; Distant/out-of-field; Broad escape | Harmonized as involvement endpoints; categories may overlap and should not be summed. | The recurrence table reports 30 central, 6 in-field, 16 marginal, and 16 distant recurrences among 68 enhancing failures; the sheet correctly combines central+in-field as 36 local and marginal+distant as 32 nonlocal. \| Escape: Sensitivity column copied from n_nonlocal_involv_enhancing for rows already reporting a nonlocal component. |
| **Sheu et al. 2019** | 2019; United States; Retrospective | Analysis stratum: Upfront first progression; arm descriptor: None/upfront | Unclear/NR; Dose-based failure; progression: NR | Local: 58/67; Marginal: 4/67; Distant: 8/67; Broad escape: 12/67; Non-enhancing/FLAIR: NR | Local/in-field; Marginal/field-edge; Distant/out-of-field; Broad escape | Harmonized as involvement endpoints; categories may overlap and should not be summed. | Across IMRT and VMAT groups there were 58 local failures, 4 regional failures, 5 distant-only failures, and 3 multifocal local+distant failures; the sheet's involvement counts are correct. \| Escape: Sensitivity column copied from n_nonlocal_involv_enhancing for rows already reporting a nonlocal component. |
| **Faustino et al. 2020** | 2020; Brazil; Retrospective | Analysis stratum: Upfront first progression; arm descriptor: None/upfront | Dose/volume based; Dose-based failure; progression: NR | Local: 34/41; Marginal: 4/41; Distant: 3/41; Broad escape: 7/41; Non-enhancing/FLAIR: NR | Local/in-field; Marginal/field-edge; Distant/out-of-field; Broad escape | Harmonized as involvement endpoints; categories may overlap and should not be summed. | Forty-one failures were classified as 34 in-field, 4 marginal, and 3 distant. \| Escape: Sensitivity column copied from n_nonlocal_involv_enhancing for rows already reporting a nonlocal component. |
| **Fleischmann et al. 2020** | 2020; Germany; Retrospective | Analysis stratum: Upfront first progression; arm descriptor: None/upfront | Dose/volume based; Dose-based failure; progression: RANO or Macdonald | Local: 34/36; Marginal: 0/36; Distant: 2/36; Broad escape: 2/36; Non-enhancing/FLAIR: NR | Local/in-field; Marginal/field-edge; Distant/out-of-field; Broad escape | Harmonized as involvement endpoints; categories may overlap and should not be summed. | For the PET-MRGTV plus 15 mm CTV margin analysis, recurrence was 32 central, 2 in-field, 0 marginal, and 2 ex-field among 36 patients; the sheet correctly combines central+in-field as 34 local. \| Escape: Sensitivity column copied from n_nonlocal_involv_enhancing for rows already reporting a nonlocal component. |
| **Jiang et al. 2020** | 2020; China; Retrospective | Analysis stratum: Upfront first progression; arm descriptor: None/upfront | Anatomic/descriptor based; Anatomic contact pattern; progression: RANO or Macdonald | Local: 186/247; Marginal: NR; Distant: 15/247; Broad escape: 61/247; Non-enhancing/FLAIR: NR | Local/in-field; Distant/out-of-field; Broad escape | Harmonized as involvement endpoints; categories may overlap and should not be summed. | Progression pattern classification reports 186 local, 15 distant, 33 subependymal, and 13 leptomeningeal events; nonlocal total is 61. \| Escape: Sensitivity column copied from n_nonlocal_involv_enhancing for rows already reporting a nonlocal component. |
| **Kazerooni et al. 2020** | 2020; United States; Retrospective | Analysis stratum: Upfront first progression; arm descriptor: None/upfront | Anatomic/descriptor based; Enhancing vs non-enhancing; progression: RANO or Macdonald | Local: 63/80; Marginal: NR; Distant: 17/80; Broad escape: 17/80; Non-enhancing/FLAIR: NR | Local/in-field; Distant/out-of-field; Broad escape | Harmonized as involvement endpoints; categories may overlap and should not be summed. | Across both institutions, 63 patients had near recurrence and 17 had distant recurrence; the sheet's local and distant/nonlocal counts match exactly. \| Escape: Sensitivity column copied from n_nonlocal_involv_enhancing for rows already reporting a nonlocal component. |
| **Sarria et al. 2020** | 2020; Multinational; Retrospective | Analysis stratum: Upfront first progression; arm descriptor: None/upfront | Dose/volume based; Dose-based failure; progression: RANO or Macdonald | Local: 18/36; Marginal: NR; Distant: 18/36; Broad escape: 18/36; Non-enhancing/FLAIR: NR | Local/in-field; Distant/out-of-field; Broad escape | Harmonized as involvement endpoints; categories may overlap and should not be summed. | Of 51 patients, 36 progressed; the first site of progression was local in 18 and distant in 18, matching the sheet exactly. \| Escape: Sensitivity column copied from n_nonlocal_involv_enhancing for rows already reporting a nonlocal component. |
| **Yamaki et al. 2020** | 2020; Japan; Retrospective | Analysis stratum: Upfront first progression; arm descriptor: None/upfront | Anatomic/descriptor based; Anatomic contact pattern; progression: NR | Local: 105/140; Marginal: NR; Distant: 35/140; Broad escape: 35/140; Non-enhancing/FLAIR: NR | Local/in-field; Distant/out-of-field; Broad escape | Harmonized as involvement endpoints; categories may overlap and should not be summed. | Of 167 patients, 140 recurred, including 105 local recurrences and 35 distant recurrences; the sheet's local and distant/nonlocal counts match these totals. \| Escape: Sensitivity column copied from n_nonlocal_involv_enhancing for rows already reporting a nonlocal component. \| Overlap: Yamagata plus Tohoku upfront cohort (January 2009 to January 2018) likely overlaps the later Toyoda V-cohort compiled from the same collaborating institutions and may also partially overlap the later single-center Tohoku Shimoda co... |
| **Comas et al. 2021** | 2021; Spain; Retrospective | Analysis stratum: Upfront first progression; arm descriptor: None/upfront | Dose/volume based; Dose-based failure; progression: RANO or Macdonald | Local: 88/101; Marginal: 5/101; Distant: 8/101; Broad escape: 13/101; Non-enhancing/FLAIR: NR | Local/in-field; Marginal/field-edge; Distant/out-of-field; Broad escape | Harmonized as involvement endpoints; categories may overlap and should not be summed. | The T1-weighted isodose table shows 83 central, 5 in-field, 5 marginal, and 8 distal relapses among 101 radiologic progressions; focality at relapse was multifocal in 59 cases. \| Escape: Sensitivity column copied from n_nonlocal_involv_enhancing for rows already reporting a nonlocal component. |
| **Hirono et al. 2021_A** | 2021; Japan; Retrospective | Analysis stratum: Upfront first progression; arm descriptor: None/upfront | Anatomic/descriptor based; Anatomic pattern; progression: RANO | Local: 14/17; Marginal: NR; Distant: 1/17; Broad escape: 3/17; Non-enhancing/FLAIR: NR | Local/in-field; Distant/out-of-field; Broad escape | Harmonized as involvement endpoints; categories may overlap and should not be summed. | In the GTR group, there were 14 local recurrences, 1 distant recurrence, and 2 leptomeningeal dissemination events among 17 recurrence events; the sheet's local 14 and nonlocal 3 are consistent. \| Escape: Sensitivity column copied from n_nonlocal_involv_enhancing for rows already reporting a nonlocal component. |
| **Hirono et al. 2021_B** | 2021; Japan; Retrospective | Analysis stratum: Upfront first progression; arm descriptor: None/upfront | Anatomic/descriptor based; Anatomic pattern; progression: RANO | Local: 0/4; Marginal: NR; Distant: 4/4; Broad escape: 4/4; Non-enhancing/FLAIR: NR | Local/in-field; Distant/out-of-field; Broad escape | Harmonized as involvement endpoints; categories may overlap and should not be summed. | In the SupTR group, all 4 recurrence events were distant recurrences, with no local or leptomeningeal failures reported. \| Escape: Sensitivity column copied from n_nonlocal_involv_enhancing for rows already reporting a nonlocal component. |
| **Kim et al. 2021** | 2021; United States; Clinical trial | Analysis stratum: Upfront first progression; arm descriptor: None/upfront | Dose/volume based; Dose-based failure; progression: RANO or Macdonald | Local: 5/16; Marginal: NR; Distant: NR; Broad escape: 11/16; Non-enhancing/FLAIR: NR | Local/in-field; Broad escape | Harmonized as involvement endpoints; categories may overlap and should not be summed. | Among 16 progressions, only 5 were central or in-field and 69% occurred outside the high-dose boost region; the sheet's local 5 and nonlocal 11 are correct. \| Escape: Sensitivity column copied from n_nonlocal_involv_enhancing for rows already reporting a nonlocal component. |
| **Shim et al. 2021** | 2021; South Korea; Retrospective | Analysis stratum: Upfront first progression; arm descriptor: None/upfront | Anatomic/descriptor based; Enhancing vs non-enhancing; progression: RANO | Local: 93/125; Marginal: NR; Distant: 76/125; Broad escape: 76/125; Non-enhancing/FLAIR: NR | Local/in-field; Distant/out-of-field; Broad escape | Harmonized as involvement endpoints; categories may overlap and should not be summed. | source confirmed after pdf remap \| Corrected PDF to Schim 2021; source gives 49 local, 32 distant, and 44 combined recurrences, supporting local 93 and distant 76 involvement counts. \| Escape: Sensitivity column copied from n_nonlocal_involv_enhancing for rows already reporting a nonlocal component. |
| **Ali et al. 2022** | 2022; United States; Clinical trial | Analysis stratum: Upfront first progression; arm descriptor: None/upfront | Distance based; Dose-based failure; progression: RANO | Local: 18/27; Marginal: NR; Distant: 6/27; Broad escape: 6/27; Non-enhancing/FLAIR: NR | Local/in-field; Distant/out-of-field; Broad escape | Harmonized as involvement endpoints; categories may overlap and should not be summed. | Of 27 patients with imaging-reviewed progression, 18 recurred within 2 cm and 6 were distant; 2 additional progressions lacked review imaging and remain outside the pattern denominator. \| Escape: Sensitivity column copied from n_nonlocal_involv_enhancing for rows already reporting a nonlocal component. |
| **Glas et al. 2022_A** | 2022; Multinational; Post hoc | Analysis stratum: Upfront first progression; arm descriptor: None/upfront | Dose/volume based; Dose-based failure; progression: NR | Local: 235/306; Marginal: NR; Distant: 71/306; Broad escape: 71/306; Non-enhancing/FLAIR: NR | Local/in-field; Distant/out-of-field; Broad escape | Harmonized as involvement endpoints; categories may overlap and should not be summed. | In the TTFields plus TMZ progression analysis, 235 patients had local and 71 had non-local progression among 306 evaluable patients; the sheet matches these totals. \| Escape: Sensitivity column copied from n_nonlocal_involv_enhancing for rows already reporting a nonlocal component. |
| **Glas et al. 2022_B** | 2022; Multinational; Post hoc | Analysis stratum: Upfront first progression; arm descriptor: None/upfront | Dose/volume based; Dose-based failure; progression: NR | Local: 101/122; Marginal: NR; Distant: 21/122; Broad escape: 21/122; Non-enhancing/FLAIR: NR | Local/in-field; Distant/out-of-field; Broad escape | Harmonized as involvement endpoints; categories may overlap and should not be summed. | In the TMZ-alone progression analysis, 101 patients had local and 21 had non-local progression among 122 evaluable patients; the sheet matches these totals. \| Escape: Sensitivity column copied from n_nonlocal_involv_enhancing for rows already reporting a nonlocal component. |
| **Yoo et al. 2022_A** | 2022; South Korea; Retrospective | Analysis stratum: Upfront first progression; arm descriptor: None/upfront | Dose/volume based; Dose-based failure; progression: RANO or Macdonald | Local: 15/26; Marginal: 3/26; Distant: 13/26; Broad escape: NR; Non-enhancing/FLAIR: NR | Local/in-field; Marginal/field-edge; Distant/out-of-field | Harmonized as involvement endpoints; categories may overlap and should not be summed. | For the SupTR subgroup, Table 2 reports 26 recurrences with 15 local, 3 marginal, and 13 distant events; because recurrence patterns overlapped across categories, the nonlocal union was left missing rather than summing marginal and distant. \| Escape: Left blank intentionally: local, marginal, and distant recurrence patterns overlap within the SupTR subgroup, so the escape-pattern union is not uniquely derivable. \| Overlap: Same Yonsei/Severance institutional system and overlapping upfront recurrence window as Ch... |
| **Yoo et al. 2022_B** | 2022; South Korea; Retrospective | Analysis stratum: Upfront first progression; arm descriptor: None/upfront | Dose/volume based; Dose-based failure; progression: RANO or Macdonald | Local: 111/146; Marginal: 8/146; Distant: 44/146; Broad escape: NR; Non-enhancing/FLAIR: NR | Local/in-field; Marginal/field-edge; Distant/out-of-field | Harmonized as involvement endpoints; categories may overlap and should not be summed. | For the GTR subgroup, Table 2 reports 146 recurrences with 111 local, 8 marginal, and 44 distant events; because recurrence patterns overlapped across categories, the nonlocal union was left missing rather than summing marginal and distant. \| Escape: Left blank intentionally: local, marginal, and distant recurrence patterns overlap within the GTR subgroup, so the escape-pattern union is not uniquely derivable. \| Overlap: Same Yonsei/Severance institutional system and overlapping upfront recurrence window as Choi... |
| **Yoo et al. 2022_C** | 2022; South Korea; Retrospective | Analysis stratum: Upfront first progression; arm descriptor: None/upfront | Dose/volume based; Dose-based failure; progression: RANO or Macdonald | Local: 82/99; Marginal: 7/99; Distant: 23/99; Broad escape: NR; Non-enhancing/FLAIR: NR | Local/in-field; Marginal/field-edge; Distant/out-of-field | Harmonized as involvement endpoints; categories may overlap and should not be summed. | For the STR subgroup, Table 2 reports 99 recurrences with 82 local, 7 marginal, and 23 distant events; because recurrence patterns overlapped across categories, the nonlocal union was left missing rather than summing marginal and distant. \| Escape: Left blank intentionally: local, marginal, and distant recurrence patterns overlap within the STR subgroup, so the escape-pattern union is not uniquely derivable. \| Overlap: Same Yonsei/Severance institutional system and overlapping upfront recurrence window as Choi e... |
| **Demircan et al. 2023** | 2023; Turkey; Retrospective | Analysis stratum: Upfront first progression; arm descriptor: None/upfront | Dose/volume based; Dose-based failure; progression: RANO or Macdonald | Local: 145/156; Marginal: 6/156; Distant: 14/156; Broad escape: 20/156; Non-enhancing/FLAIR: NR | Local/in-field; Marginal/field-edge; Distant/out-of-field; Broad escape | Harmonized as involvement endpoints; categories may overlap and should not be summed. | Among 156 recurrent/progressive lesions, 33% were central, 54% in-field, 4% marginal, 3% out-of-field, and 6% both in and out of field; the sheet's local 145 and nonlocal 20 preserve overlap. \| Escape: Sensitivity column copied from n_nonlocal_involv_enhancing for rows already reporting a nonlocal component. |
| **Guberina et al. 2023** | 2023; Germany; Retrospective | Analysis stratum: Upfront first progression; arm descriptor: None/upfront | Dose/volume based; Dose-based failure; progression: RANO or Macdonald | Local: 80/91; Marginal: 5/91; Distant: 11/91; Broad escape: 16/91; Non-enhancing/FLAIR: NR | Local/in-field; Marginal/field-edge; Distant/out-of-field; Broad escape | Harmonized as involvement endpoints; categories may overlap and should not be summed. | Recurrence geometry shows 80 in-field within the 95% isodose, 5 marginal, 11 out-field, and 17 multifocal recurrences. \| Escape: Sensitivity column copied from n_nonlocal_involv_enhancing for rows already reporting a nonlocal component. |
| **Liu et al. 2023** | 2023; China; Retrospective | Analysis stratum: Upfront first progression; arm descriptor: None/upfront | Dose/volume based; Dose-based failure; progression: RANO or Macdonald | Local: 52/66; Marginal: 0/66; Distant: 8/66; Broad escape: 14/66; Non-enhancing/FLAIR: NR | Local/in-field; Marginal/field-edge; Distant/out-of-field; Broad escape | Harmonized as involvement endpoints; categories may overlap and should not be summed. | The GBM subgroup table (n=66 relapses) shows 45 central, 7 in-field, 0 marginal, 8 distant, and 6 CSF-d events; reclassified local/nonlocal totals of 52 and 14 match the sheet. \| Escape: Sensitivity column copied from n_nonlocal_involv_enhancing for rows already reporting a nonlocal component. |
| **Mendoza et al. 2023** | 2023; United States; Prospective | Analysis stratum: Upfront first progression; arm descriptor: None/upfront | Dose/volume based; Dose-based failure; progression: RANO or Macdonald | Local: 17/27; Marginal: 3/27; Distant: 8/27; Broad escape: 11/27; Non-enhancing/FLAIR: NR | Local/in-field; Marginal/field-edge; Distant/out-of-field; Broad escape | Harmonized as involvement endpoints; categories may overlap and should not be summed. | Among 27 progressions, 17 were in-field, 3 marginal, and 7 distant, with one simultaneous in-field+distant case; the sheet's involvement-based distant count of 8 is correct. \| Escape: Sensitivity column copied from n_nonlocal_involv_enhancing for rows already reporting a nonlocal component. |
| **Minniti et al. 2023** | 2023; Italy; Retrospective | Analysis stratum: Upfront first progression; arm descriptor: None/upfront | Dose/volume based; Dose-based failure; progression: RANO or Macdonald | Local: 184/207; Marginal: 5/207; Distant: 22/207; Broad escape: 27/207; Non-enhancing/FLAIR: NR | Local/in-field; Marginal/field-edge; Distant/out-of-field; Broad escape | Harmonized as involvement endpoints; categories may overlap and should not be summed. | The paper reports 180 in-field, 5 marginal, and 22 distant recurrences and notes 4 simultaneous in-field+distant cases; the sheet retains involvement-based local/nonlocal totals to preserve overlap. \| Escape: Sensitivity column copied from n_nonlocal_involv_enhancing for rows already reporting a nonlocal component. \| Overlap: University of Siena and linked Neuromed/UPMC cohort from February 2015 to July 2020 likely overlaps substantially with Tini et al. 2025 (January 2016 to February 2024) and may also share a... |
| **Mizuhata et al. 2023_A** | 2023; Japan; Retrospective | Analysis stratum: Upfront first progression; arm descriptor: None/upfront | Anatomic/descriptor based; Dose-based failure; progression: RANO | Local: 30/34; Marginal: NR; Distant: 5/34; Broad escape: 5/34; Non-enhancing/FLAIR: NR | Local/in-field; Distant/out-of-field; Broad escape | Harmonized as involvement endpoints; categories may overlap and should not be summed. | Standard-course arm: 29 local, 4 distant, and 1 local+distant recurrence among 34 patients; the sheet correctly encodes local 30 and distant/nonlocal 5. \| Escape: Sensitivity column copied from n_nonlocal_involv_enhancing for rows already reporting a nonlocal component. |
| **Mizuhata et al. 2023_B** | 2023; Japan; Retrospective | Analysis stratum: Upfront first progression; arm descriptor: None/upfront | Anatomic/descriptor based; Dose-based failure; progression: RANO | Local: 21/24; Marginal: NR; Distant: 5/24; Broad escape: 5/24; Non-enhancing/FLAIR: NR | Local/in-field; Distant/out-of-field; Broad escape | Harmonized as involvement endpoints; categories may overlap and should not be summed. | Short-course arm: 20 local, 4 distant, and 1 local+distant recurrence among 24 patients; the sheet correctly encodes local 21 and distant/nonlocal 5. \| Escape: Sensitivity column copied from n_nonlocal_involv_enhancing for rows already reporting a nonlocal component. |
| **Noeuveglise et al. 2023** | 2023; France; Retrospective | Analysis stratum: Upfront first progression; arm descriptor: None/upfront | Anatomic/descriptor based; Dose-based failure; progression: NR | Local: 81/128; Marginal: 34/128; Distant: 13/128; Broad escape: 47/128; Non-enhancing/FLAIR: NR | Local/in-field; Marginal/field-edge; Distant/out-of-field; Broad escape | Harmonized as involvement endpoints; categories may overlap and should not be summed. | The paper defines 34 marginal relapses by overlap score <=0.95 and 13 out-field relapses by overlap score <0.8; the remaining 81 relapses are in-field, matching the sheet. \| Escape: Sensitivity column copied from n_nonlocal_involv_enhancing for rows already reporting a nonlocal component. |
| **Di Perri et al. 2024** | 2024; Netherlands; Retrospective | Analysis stratum: Upfront first progression; arm descriptor: None/upfront | Dose/volume based; Dose-based failure; progression: Macdonald | Local: 67/75; Marginal: 6/75; Distant: 12/75; Broad escape: 16/75; Non-enhancing/FLAIR: NR | Local/in-field; Marginal/field-edge; Distant/out-of-field; Broad escape | Harmonized as involvement endpoints; categories may overlap and should not be summed. | Combined cohorts yield 67 in-field, 6 marginal, and 12 distant-involvement recurrences, including simultaneous distant lesions. \| Escape: Sensitivity column copied from n_nonlocal_involv_enhancing for rows already reporting a nonlocal component. |
| **Senyurek et al. 2024** | 2024; Turkey; Retrospective | Analysis stratum: Upfront first progression; arm descriptor: None/upfront | Dose/volume based; GTV-based failure; progression: RANO or Macdonald | Local: 49/71; Marginal: 4/71; Distant: 27/71; Broad escape: 31/71; Non-enhancing/FLAIR: NR | Local/in-field; Marginal/field-edge; Distant/out-of-field; Broad escape | Harmonized as involvement endpoints; categories may overlap and should not be summed. | Among 71 failures, 40 were intra-GTV, 4 in-CTV/out-GTV, 18 distant, and 9 intra-GTV+distant; the sheet correctly encodes local 49, marginal 4, and distant/nonlocal 27. \| Escape: Sensitivity column copied from n_nonlocal_involv_enhancing for rows already reporting a nonlocal component. |
| **Toyoda et al. 2024_A** | 2024; Japan; Retrospective | Analysis stratum: Upfront first progression; arm descriptor: None/upfront | Anatomic/descriptor based; Anatomic contact pattern; progression: RANO or Macdonald | Local: 56/77; Marginal: NR; Distant: NR; Broad escape: 21/77; Non-enhancing/FLAIR: NR | Local/in-field; Broad escape | Harmonized as involvement endpoints; categories may overlap and should not be summed. | The K-cohort had 77 first recurrences with 56 local and 21 non-local events; 6 additional patients had no recurrence or were lost to follow-up, matching the sheet's denominators. \| Escape: Sensitivity column copied from n_nonlocal_involv_enhancing for rows already reporting a nonlocal component. |
| **Braschi et al. 2025** | 2025; United States; Retrospective | Analysis stratum: Upfront first progression; arm descriptor: None/upfront | Dose/volume based; Dose-based failure; progression: RANO or Macdonald | Local: 45/50; Marginal: 1/50; Distant: 9/50; Broad escape: 10/50; Non-enhancing/FLAIR: NR | Local/in-field; Marginal/field-edge; Distant/out-of-field; Broad escape | Harmonized as involvement endpoints; categories may overlap and should not be summed. | In-field alone 40, in-field+distant 5, distant alone 4, marginal alone 1; multisite recurrence count 14 confirmed. \| Escape: Sensitivity column copied from n_nonlocal_involv_enhancing for rows already reporting a nonlocal component. |
| **Crompton et al. 2025_A** | 2025; United States; Retrospective | Analysis stratum: Upfront first progression; arm descriptor: None/upfront | Dose/volume based; Dose-based failure; progression: NR | Local: 201/226; Marginal: 14/226; Distant: 11/226; Broad escape: 25/226; Non-enhancing/FLAIR: NR | Local/in-field; Marginal/field-edge; Distant/out-of-field; Broad escape | Harmonized as involvement endpoints; categories may overlap and should not be summed. | In the non-TTF cohort, location of failure was 201 in-field, 14 marginal, and 11 distant among 226 progressions; the sheet matches these counts. \| Escape: Sensitivity column copied from n_nonlocal_involv_enhancing for rows already reporting a nonlocal component. |
| **Crompton et al. 2025_B** | 2025; United States; Retrospective | Analysis stratum: Upfront first progression; arm descriptor: None/upfront | Dose/volume based; Dose-based failure; progression: NR | Local: 49/58; Marginal: 8/58; Distant: 1/58; Broad escape: 9/58; Non-enhancing/FLAIR: NR | Local/in-field; Marginal/field-edge; Distant/out-of-field; Broad escape | Harmonized as involvement endpoints; categories may overlap and should not be summed. | In the TTF cohort, location of failure was 49 in-field, 8 marginal, and 1 distant among 58 progressions; the sheet matches these counts. \| Escape: Sensitivity column copied from n_nonlocal_involv_enhancing for rows already reporting a nonlocal component. |
| **Fujimoto et al. 2025_A** | 2025; Japan; Retrospective | Analysis stratum: Upfront first progression; arm descriptor: None/upfront | Anatomic/descriptor based; Anatomic contact pattern; progression: RANO | Local: 22/39; Marginal: NR; Distant: 19/39; Broad escape: 19/39; Non-enhancing/FLAIR: NR | Local/in-field; Distant/out-of-field; Broad escape | Harmonized as involvement endpoints; categories may overlap and should not be summed. | PDT arm: 20 local, 8 distant, 9 dissemination, 2 local+distant/dissemination; involvement-based local 22 and nonlocal 19 confirmed. \| Escape: Sensitivity column copied from n_nonlocal_involv_enhancing for rows already reporting a nonlocal component. |
| **Fujimoto et al. 2025_B** | 2025; Japan; Retrospective | Analysis stratum: Upfront first progression; arm descriptor: None/upfront | Anatomic/descriptor based; Anatomic contact pattern; progression: RANO | Local: 48/56; Marginal: NR; Distant: 17/56; Broad escape: 17/56; Non-enhancing/FLAIR: NR | Local/in-field; Distant/out-of-field; Broad escape | Harmonized as involvement endpoints; categories may overlap and should not be summed. | Non-PDT arm: 40 local, 3 distant, 6 dissemination, 8 local+distant/dissemination; involvement-based local 48 and nonlocal 17 confirmed. \| Escape: Sensitivity column copied from n_nonlocal_involv_enhancing for rows already reporting a nonlocal component. |
| **Laviv et al. 2025** | 2025; Israel; Retrospective | Analysis stratum: Upfront first progression; arm descriptor: None/upfront | Anatomic/descriptor based; Anatomic contact pattern; progression: Macdonald | Local: NR; Marginal: NR; Distant: 7/94; Broad escape: 7/94; Non-enhancing/FLAIR: NR | Distant/out-of-field; Broad escape | Harmonized as involvement endpoints; categories may overlap and should not be summed. | Table 1 reports distant recurrence in 7.45% of the 94-patient cohort, which corresponds to 7 patients; the sheet's distant/nonlocal count matches this extracted field. \| Escape: Sensitivity column copied from n_nonlocal_involv_enhancing for rows already reporting a nonlocal component. |
| **Moore-Palhares et al. 2025** | 2025; Canada; Prospective | Analysis stratum: Upfront first progression; arm descriptor: None/upfront | Dose/volume based; GTV-based failure; progression: RANO | Local: 63/73; Marginal: 6/73; Distant: 4/73; Broad escape: 10/73; Non-enhancing/FLAIR: NR | Local/in-field; Marginal/field-edge; Distant/out-of-field; Broad escape | Harmonized as involvement endpoints; categories may overlap and should not be summed. | source confirmed after pdf remap \| Confirmed against 2025 ADC recurrence paper: 49 entire GTV + 14 partial = 63 local; 6 within CTV/T2-FLAIR and 4 outside CTV = 10 nonlocal. \| Escape: Sensitivity column copied from n_nonlocal_involv_enhancing for rows already reporting a nonlocal component. \| Overlap: Sunnybrook/Odette/University of Toronto prospective imaging cohort likely shares patients with the Stewart local database during the overlapping 2017 to 2019 period. Retained as the most comprehensive Sunnybrook/Un... |
| **Takido et al. 2025** | 2025; Japan; Retrospective | Analysis stratum: Upfront first progression; arm descriptor: None/upfront | Anatomic/descriptor based; Dose-based failure; progression: NR | Local: 12/15; Marginal: NR; Distant: 5/15; Broad escape: 5/15; Non-enhancing/FLAIR: NR | Local/in-field; Distant/out-of-field; Broad escape | Harmonized as involvement endpoints; categories may overlap and should not be summed. | Among 15 recurrences during continued TTFields, Table 3 shows distant parenchymal recurrence in 5 and local recurrence around the primary lesion in 12, including 2 cases with both local and distant involvement; one separate leptomeningeal case was not counted as distant parenchymal recurrence. \| Escape: Sensitivity column copied from n_nonlocal_involv_enhancing for rows already reporting a nonlocal component. |
| **Tian et al. 2025** | 2025; China; Retrospective | Analysis stratum: Upfront first progression; arm descriptor: None/upfront | Distance based; Dose-based failure; progression: RANO | Local: 23/33; Marginal: 7/33; Distant: 3/33; Broad escape: 10/33; Non-enhancing/FLAIR: NR | Local/in-field; Marginal/field-edge; Distant/out-of-field; Broad escape | Harmonized as involvement endpoints; categories may overlap and should not be summed. | The recurrence analysis included 33 patients with 23 local, 7 marginal, and 3 distant failures; the sheet's counts match exactly. \| Escape: Sensitivity column copied from n_nonlocal_involv_enhancing for rows already reporting a nonlocal component. |
| **Matsuyama et al. 2026** | 2026; Japan; Retrospective | Analysis stratum: Upfront first progression; arm descriptor: None/upfront | Dose/volume based; Dose-based failure; progression: RANO or Macdonald | Local: 32/37; Marginal: 0/37; Distant: 5/37; Broad escape: 5/37; Non-enhancing/FLAIR: NR | Local/in-field; Marginal/field-edge; Distant/out-of-field; Broad escape | Harmonized as involvement endpoints; categories may overlap and should not be summed. | The recurrence table reports 29 central, 3 in-field, 0 marginal, and 5 distant recurrences; these table counts sum to 37 despite the results text stating 36 recurrences, so the table values were used for the spatial extraction. \| Escape: Sensitivity column copied from n_nonlocal_involv_enhancing for rows already reporting a nonlocal component. |
| **Norden et al. 2008** | 2008; United States; Retrospective | Analysis stratum: Post-BEV failure; arm descriptor: BEV | Anatomic/descriptor based; Enhancing vs non-enhancing; progression: Macdonald | Local: 16/24; Marginal: NR; Distant: 4/24; Broad escape: 8/24; Non-enhancing/FLAIR: NR | Local/in-field; Distant/out-of-field; Broad escape | Harmonized as involvement endpoints; categories may overlap and should not be summed. | Among 26 reviewed bevacizumab cases, 16 were local, 4 diffuse, and 4 distant; 2 had no radiographic progression at publication and remain outside the enhancing denominator. \| Escape: Sensitivity-only composite: 4 diffuse + 4 distant progression patterns among 24 radiographic progressors. |
| **Iwamoto et al. 2009** | 2009; United States; Retrospective | Analysis stratum: Post-BEV failure; arm descriptor: Mixed/other | Anatomic/descriptor based; Enhancing vs non-enhancing; progression: Macdonald | Local: 17/36; Marginal: NR; Distant: NR; Broad escape: 19/36; Non-enhancing/FLAIR: 13/36 | Local/in-field; Broad escape; Non-enhancing/FLAIR | Harmonized as involvement endpoints; categories may overlap and should not be summed. | Source reports 17 local, 6 multifocal, 13 predominantly nonenhancing; one clinical-only progression remains outside enhancing denominator. \| Escape: Sensitivity-only composite: 6 multifocal enhancing recurrences + 13 predominantly nonenhancing progressions among the 36 radiographic progressors. \| Overlap: MSKCC recurrent GBM post-BEV failure cohort (October 2006 to January 2009) may partially overlap the later MSKCC surgical or autopsy subset reported by Thomas et al., but the latter does not report a reconstruc... |
| **Chamberlain et al. 2011_B** | 2011; United States; Retrospective | Analysis stratum: Post-BEV failure; arm descriptor: BEV | Anatomic/descriptor based; Enhancing vs non-enhancing; progression: Macdonald | Local: 57/80; Marginal: NR; Distant: 7/80; Broad escape: 23/80; Non-enhancing/FLAIR: NR | Local/in-field; Distant/out-of-field; Broad escape | Harmonized as involvement endpoints; categories may overlap and should not be summed. | source confirmed after pdf remap \| Corrected PDF to Chamberlain case report; second recurrence table confirms 57 local, 7 distant, 7 multifocal, 9 diffuse. \| Escape: Sensitivity-only composite: second recurrence table gives 9 diffuse + 7 distant + 7 multifocal mutually exclusive escape-pattern events. \| Overlap: Same 80-patient University of Washington cohort contributes a first-progression row and a later post-BEV row in the same paper; do not treat these rows as independent in any pooled model spanning stages. |
| **Pope et al. 2011_A** | 2011; United States; Post hoc | Analysis stratum: Post-BEV failure; arm descriptor: BEV | Distance based; Enhancing vs non-enhancing; progression: Macdonald | Local: 48/67; Marginal: NR; Distant: 1/67; Broad escape: 19/67; Non-enhancing/FLAIR: NR | Local/in-field; Distant/out-of-field; Broad escape | Harmonized as involvement endpoints; categories may overlap and should not be summed. | source confirmed numeric correction \| Diffuse progression count restored from source table: 14 of 67 progressors in the BEV arm. \| Escape: Sensitivity-only composite: 14 diffuse + 1 distant + 4 multifocal progression patterns among 67 BEV-arm progressors. |
| **Pope et al. 2011_B** | 2011; United States; Post hoc | Analysis stratum: Post-BEV failure; arm descriptor: BEV | Distance based; Enhancing vs non-enhancing; progression: Macdonald | Local: 40/57; Marginal: NR; Distant: 0/57; Broad escape: 17/57; Non-enhancing/FLAIR: NR | Local/in-field; Distant/out-of-field; Broad escape | Harmonized as involvement endpoints; categories may overlap and should not be summed. | source confirmed numeric correction \| Diffuse progression count restored from source table: 12 of 57 progressors in the BEV+CPT-11 arm. \| Escape: Sensitivity-only composite: 12 diffuse + 5 multifocal progression patterns among 57 BEV+CPT-11-arm progressors; no distant events reported. |
| **Desjardins et al. 2012** | 2012; United States; Clinical trial | Analysis stratum: Post-BEV failure; arm descriptor: BEV | Anatomic/descriptor based; Enhancing vs non-enhancing; progression: Macdonald | Local: 8/21; Marginal: NR; Distant: 1/21; Broad escape: 8/21; Non-enhancing/FLAIR: 5/21 | Local/in-field; Distant/out-of-field; Broad escape; Non-enhancing/FLAIR | Harmonized as involvement endpoints; categories may overlap and should not be summed. | Table 3 supports enhancing-involved counts 8 local, 7 diffuse, 1 distant with 5 nonenhancing-only events. \| Escape: Sensitivity-only composite: 7 diffuse (enhancing and nonenhancing) + 1 distant (enhancing and nonenhancing) from Table 3; nonenhancing-only rows excluded. |
| **Perez-Larraya et al. 2012** | 2012; France; Retrospective | Analysis stratum: Post-BEV failure; arm descriptor: BEV | Unclear/NR; Enhancing vs non-enhancing; progression: RANO or Macdonald | Local: 28/58; Marginal: NR; Distant: 10/58; Broad escape: 10/58; Non-enhancing/FLAIR: 20/58 | Local/in-field; Distant/out-of-field; Broad escape; Non-enhancing/FLAIR | Harmonized as involvement endpoints; categories may overlap and should not be summed. | RANO progression patterns are 28 local enhancing, 10 distant enhancing, and 20 diffuse nonenhancing among 58 progressors. \| Escape: Sensitivity column copied from n_nonlocal_involv_enhancing for rows already reporting a nonlocal component. |
| **Bahr et al. 2014** | 2014; Germany; Mixed | Analysis stratum: Post-BEV failure; arm descriptor: BEV | Anatomic/descriptor based; Enhancing vs non-enhancing; progression: RANO or Macdonald | Local: 32/61; Marginal: NR; Distant: 15/61; Broad escape: 29/61; Non-enhancing/FLAIR: NR | Local/in-field; Distant/out-of-field; Broad escape | Harmonized as involvement endpoints; categories may overlap and should not be summed. | source confirmed numeric correction \| Source table confirms 32 local, 14 diffuse, and 15 distant progression patterns among 61 progressors; diffuse count was missing in the sheet. \| Escape: Sensitivity-only composite: 14 diffuse + 15 distant progression patterns among 61 progressors. |
| **Soffietti et al. 2014** | 2014; Italy; Clinical trial | Analysis stratum: Post-BEV failure; arm descriptor: BEV | Anatomic/descriptor based; Enhancing vs non-enhancing; progression: RANO or Macdonald | Local: 34/42; Marginal: NR; Distant: 5/42; Broad escape: 9/42; Non-enhancing/FLAIR: 4/42 | Local/in-field; Distant/out-of-field; Broad escape; Non-enhancing/FLAIR | Harmonized as involvement endpoints; categories may overlap and should not be summed. | Unifocal baseline subgroup: 32 local, 3 distant, 2 multifocal, 4 diffuse nonenhancing, 1 leptomeningeal; sheet keeps enhancing involvement plus NE. \| Escape: Sensitivity-only composite: 5 distant-involved enhancing events (3 distant plus 2 multifocal local+distant) + 4 diffuse nonenhancing events in the unifocal baseline subgroup; isolated leptomeningeal spread excluded. |
| **Mamo et al. 2016** | 2016; Canada; Retrospective | Analysis stratum: Post-BEV failure; arm descriptor: BEV | Anatomic/descriptor based; Anatomic pattern; progression: NR | Local: 13/34; Marginal: NR; Distant: 5/34; Broad escape: 24/34; Non-enhancing/FLAIR: NR | Local/in-field; Distant/out-of-field; Broad escape | Harmonized as involvement endpoints; categories may overlap and should not be summed. | Second-line+ arm uses overlap counting: 9 local alone plus 4 local+distant gives local 13; 1 distant alone plus 4 mixed gives distant 5. \| Escape: Sensitivity-only composite for the B2L+ row: 16 diffuse + 3 multifocal + 5 distant-involved recurrences, keeping the main nonlocal field untouched. |
| **Cachia et al. 2017** | 2017; United States; Retrospective | Analysis stratum: Post-BEV failure; arm descriptor: BEV | Anatomic/descriptor based; Enhancing vs non-enhancing; progression: RANO or Macdonald | Local: 35/64; Marginal: NR; Distant: 14/64; Broad escape: 29/64; Non-enhancing/FLAIR: NR | Local/in-field; Distant/out-of-field; Broad escape | Harmonized as involvement endpoints; categories may overlap and should not be summed. | source confirmed numeric correction \| Baseline pre-BEV pattern table confirms local 35, diffuse 15, and distant 14; diffuse and distant had been transposed in the sheet. \| Escape: Sensitivity-only composite: 15 diffuse + 14 distant from the source local/diffuse/distant pattern table. |
| **Park et al. 2020** | 2020; South Korea; Retrospective | Analysis stratum: Post-BEV failure; arm descriptor: BEV | Distance based; Enhancing vs non-enhancing; progression: RANO or Macdonald | Local: 20/43; Marginal: NR; Distant: NR; Broad escape: 23/43; Non-enhancing/FLAIR: 23/43 | Local/in-field; Broad escape; Non-enhancing/FLAIR | Harmonized as involvement endpoints; categories may overlap and should not be summed. | Progression cohort of 43 splits into 20 local enhancing and 23 diffuse nonenhancing recurrences. \| Escape: Sensitivity-only composite: 23 diffuse nonenhancing recurrences in the 43-patient bevacizumab progression cohort. \| Overlap: Same Asan Medical Center bevacizumab-treated recurrent GBM population as the Moon bevacizumab arm, with overlapping accrual during 2017 to 2019. Retained as the most comprehensive Asan Medical Center bevacizumab cohort after exclusion of the overlapping Moon bevacizumab arm. |
| **Ciammella et al. 2013** | 2013; Italy; Retrospective | Analysis stratum: Post-reRT failure; arm descriptor: reRT | Unclear/NR; Dose-based failure; progression: NR | Local: 61/83; Marginal: 7/83; Distant: 15/83; Broad escape: 22/83; Non-enhancing/FLAIR: NR | Local/in-field; Marginal/field-edge; Distant/out-of-field; Broad escape | Harmonized as involvement endpoints; categories may overlap and should not be summed. | Final source audit confirmed source text: pattern of recurrences analyzed in 83 patients; 61 in-field, 7 at RT field margin, and 15 out-field. No numerator/denominator change. \| Escape: Sensitivity column copied from n_nonlocal_involv_enhancing for rows already reporting a nonlocal component. |
| **Niyazi et al. 2014** | 2014; Germany; Retrospective | Analysis stratum: Post-reRT failure; arm descriptor: Mixed/other | Dose/volume based; Dose-based failure; progression: RANO or Macdonald | Local: 19/31; Marginal: 7/31; Distant: 5/31; Broad escape: 12/31; Non-enhancing/FLAIR: NR | Local/in-field; Marginal/field-edge; Distant/out-of-field; Broad escape | Harmonized as involvement endpoints; categories may overlap and should not be summed. | Recurrence patterns were 19 in-field, 7 marginal, and 5 ex-field after re-irradiation with bevacizumab. \| Escape: Sensitivity column copied from n_nonlocal_involv_enhancing for rows already reporting a nonlocal component. |
| **Straube et al. 2017** | 2017; Germany; Retrospective | Analysis stratum: Post-reRT failure; arm descriptor: Surgery/local | Dose/volume based; Dose-based failure; progression: RANO or Macdonald | Local: 16/20; Marginal: NR; Distant: 6/20; Broad escape: 6/20; Non-enhancing/FLAIR: NR | Local/in-field; Distant/out-of-field; Broad escape | Harmonized as involvement endpoints; categories may overlap and should not be summed. | After gross-total resection of recurrent GBM, 14 of 20 failures were local, 2 local+distant, and 4 distant only; the sheet's local 16 and nonlocal 6 are correct. \| Escape: Sensitivity column copied from n_nonlocal_involv_enhancing for rows already reporting a nonlocal component. |
| **Ene et al. 2019** | 2019; United States; Retrospective | Analysis stratum: Post-reRT failure; arm descriptor: reRT | Dose/volume based; Dose-based failure; progression: RANO or Macdonald | Local: 40/47; Marginal: 2/47; Distant: 5/47; Broad escape: 7/47; Non-enhancing/FLAIR: NR | Local/in-field; Marginal/field-edge; Distant/out-of-field; Broad escape | Harmonized as involvement endpoints; categories may overlap and should not be summed. | Following SRS, failures were 40 in-field, 2 marginal, 2 distant, and 3 remote; the sheet correctly records 40 local, 2 marginal, and 5 distant/out-field involvement. \| Escape: Sensitivity column copied from n_nonlocal_involv_enhancing for rows already reporting a nonlocal component. |
| **Dono et al. 2021** | 2021; United States; Retrospective | Analysis stratum: Post-reRT failure; arm descriptor: reRT | Unclear/NR; Dose-based failure; progression: RANO or Macdonald | Local: 15/33; Marginal: NR; Distant: NR; Broad escape: 18/33; Non-enhancing/FLAIR: NR | Local/in-field; Broad escape | Harmonized as involvement endpoints; categories may overlap and should not be summed. | Table 1 shows 10 no recurrence, 15 in-field recurrence, and 18 out-of-field recurrence after SRS; the sheet correctly uses 33 evaluable failures with 15 local and 18 nonlocal. \| Escape: Sensitivity column copied from n_nonlocal_involv_enhancing for rows already reporting a nonlocal component. |
| **Datta et al. 2023** | 2023; India; Retrospective | Analysis stratum: Post-reRT failure; arm descriptor: reRT | Dose/volume based; Dose-based failure; progression: NR | Local: 27/37; Marginal: 4/37; Distant: 6/37; Broad escape: 10/37; Non-enhancing/FLAIR: NR | Local/in-field; Marginal/field-edge; Distant/out-of-field; Broad escape | Harmonized as involvement endpoints; categories may overlap and should not be summed. | Table 3 reports 27 central/in-field, 4 marginal, and 6 distant recurrences. \| Escape: Sensitivity column copied from n_nonlocal_involv_enhancing for rows already reporting a nonlocal component. |
| **You et al. 2023_A** | 2023; Taiwan; Retrospective | Analysis stratum: Post-reRT failure; arm descriptor: reRT | Unclear/NR; Dose-based failure; progression: RANO or Macdonald | Local: 25/35; Marginal: NR; Distant: NR; Broad escape: 13/35; Non-enhancing/FLAIR: NR | Local/in-field; Broad escape | Harmonized as involvement endpoints; categories may overlap and should not be summed. | In the non-ReRT group, bevacizumab failure was locoregional in 22, LMS in 10, and both in 3; the sheet's local 25 and nonlocal 13 preserve overlap. \| Escape: Sensitivity column copied from n_nonlocal_involv_enhancing for rows already reporting a nonlocal component. |
| **You et al. 2023_B** | 2023; Taiwan; Retrospective | Analysis stratum: Post-reRT failure; arm descriptor: reRT | Unclear/NR; Dose-based failure; progression: RANO or Macdonald | Local: 19/29; Marginal: NR; Distant: NR; Broad escape: 13/29; Non-enhancing/FLAIR: NR | Local/in-field; Broad escape | Harmonized as involvement endpoints; categories may overlap and should not be summed. | In the ReRT group, bevacizumab failure was locoregional in 16, LMS in 10, and both in 3; the sheet's local 19 and nonlocal 13 preserve overlap. \| Escape: Sensitivity column copied from n_nonlocal_involv_enhancing for rows already reporting a nonlocal component. |
| **Christ et al. 2024** | 2024; United States; Retrospective | Analysis stratum: Post-reRT failure; arm descriptor: Mixed/other | Unclear/NR; Dose-based failure; progression: RANO or Macdonald | Local: 35/89; Marginal: 28/89; Distant: NR; Broad escape: 54/89; Non-enhancing/FLAIR: NR | Local/in-field; Marginal/field-edge; Broad escape | Harmonized as involvement endpoints; categories may overlap and should not be summed. | Among 89 known failures after re-RT, 35 were local, 28 marginal, and 26 distant/multifocal; the sheet preserves local and nonlocal totals without forcing a distant-only split. \| Escape: Sensitivity column copied from n_nonlocal_involv_enhancing for rows already reporting a nonlocal component. |
| **Pepper et al. 2024** | 2024; Germany; Retrospective | Analysis stratum: Post-reRT failure; arm descriptor: reRT | Dose/volume based; Dose-based failure; progression: RANO or Macdonald | Local: 28/57; Marginal: 26/57; Distant: 3/57; Broad escape: 29/57; Non-enhancing/FLAIR: NR | Local/in-field; Marginal/field-edge; Distant/out-of-field; Broad escape | Harmonized as involvement endpoints; categories may overlap and should not be summed. | For the 57 patients with post-reRT MRI available, recurrence was 28 in-field, 26 marginal, and 3 out-field; the sheet matches this analyzed subgroup. \| Escape: Sensitivity column copied from n_nonlocal_involv_enhancing for rows already reporting a nonlocal component. |
| **Rogers et al. 2024** | 2024; Switzerland; Retrospective | Analysis stratum: Post-reRT failure; arm descriptor: reRT | Dose/volume based; Dose-based failure; progression: NR | Local: 21/28; Marginal: 2/28; Distant: 5/28; Broad escape: 7/28; Non-enhancing/FLAIR: NR | Local/in-field; Marginal/field-edge; Distant/out-of-field; Broad escape | Harmonized as involvement endpoints; categories may overlap and should not be summed. | In the MRI-followed subgroup, 28 radiological relapses were classifiable after re-mHSRT: 21 in-field, 2 marginal, and 5 out-of-field; these counts were extracted directly. \| Escape: Sensitivity column copied from n_nonlocal_involv_enhancing for rows already reporting a nonlocal component. |
| **Tong et al. 2024** | 2024; Australia; Retrospective | Analysis stratum: Post-reRT failure; arm descriptor: reRT | Dose/volume based; Dose-based failure; progression: NR | Local: 273/396; Marginal: 28/396; Distant: 131/396; Broad escape: 159/396; Non-enhancing/FLAIR: NR | Local/in-field; Marginal/field-edge; Distant/out-of-field; Broad escape | Harmonized as involvement endpoints; categories may overlap and should not be summed. | Among 396 progression events there were 237 isolated local, 28 marginal, 95 isolated distant, and 36 combined local+distant events; involvement counts in the sheet are consistent. \| Escape: Sensitivity column copied from n_nonlocal_involv_enhancing for rows already reporting a nonlocal component. |
| **Margulies et al. 2025** | 2025; France; Retrospective | Analysis stratum: Post-reRT failure; arm descriptor: reRT | Dose/volume based; Dose-based failure; progression: RANO or Macdonald | Local: 10/62; Marginal: 25/62; Distant: 27/62; Broad escape: 52/62; Non-enhancing/FLAIR: NR | Local/in-field; Marginal/field-edge; Distant/out-of-field; Broad escape | Harmonized as involvement endpoints; categories may overlap and should not be summed. | After fSRT, recurrences were 10 in-field, 25 marginal, and 27 out-field, with 21 multifocal cases; the sheet matches these counts. \| Escape: Sensitivity column copied from n_nonlocal_involv_enhancing for rows already reporting a nonlocal component. |
| **Dorner et al. 2013_B** | 2013; Germany; Prospective | Analysis stratum: Post-other salvage failure; arm descriptor: Mixed/other | Anatomic/descriptor based; Anatomic pattern; progression: NR | Local: 12/22; Marginal: NR; Distant: 3/22; Broad escape: 3/22; Non-enhancing/FLAIR: NR | Local/in-field; Distant/out-of-field; Broad escape | Harmonized as involvement endpoints; categories may overlap and should not be summed. | source confirmed numeric correction \| Recurrent-glioma subgroup (n=22) had 9 local, 10 diffuse, and 3 multilocular recurrences; the missing nonlocal-involvement count of 3 was restored. \| Escape: Sensitivity column copied from n_nonlocal_involv_enhancing for rows already reporting a nonlocal component. |
| **Moon et al. 2022_B** | 2022; South Korea; Retrospective | Analysis stratum: Post-other salvage failure; arm descriptor: Mixed/other | Unclear/NR; Enhancing vs non-enhancing; progression: RANO or Macdonald | Local: 11/16; Marginal: NR; Distant: 4/16; Broad escape: 4/16; Non-enhancing/FLAIR: 1/16 | Local/in-field; Distant/out-of-field; Broad escape; Non-enhancing/FLAIR | Harmonized as involvement endpoints; categories may overlap and should not be summed. | Temozolomide arm: 16 progressed, with 11 local enhancing, 1 diffuse nonenhancing, and 4 distant progression. \| Escape: Sensitivity column copied from n_nonlocal_involv_enhancing for rows already reporting a nonlocal component. |

**Supplementary table 6. Adapted JBI quality and risk-of-bias assessment for included study arms.**

Rating key: Y = yes/low concern for that item; U = unclear/some concern; N = no/high concern. Overall concern was calculated as Y=0, U=1, N=2; Low = 0-2, Moderate = 3-5, High >=6. The appraisal adapts the JBI prevalence checklist to recurrence-location proportion reporting; it evaluates interpretability of spatial-proportion estimates, not comparative treatment efficacy.

| **Item** | **Adapted appraisal question** | **Operational rule** |
| --- | --- | --- |
| **JBI1** | Appropriate sample frame for the target population | Y if GBM-only target population; U if mixed HGG/GBM. |
| **JBI2** | Appropriate sampling/recruitment approach | Y for clinical trial, prospective cohort, or post hoc analysis of a defined cohort; U for retrospective or mixed designs without verifiable consecutive sampling in the extracted fields. |
| **JBI3** | Adequate sample size for descriptive proportion extraction | Y if arm n >=50, U if n=30-49, N if n<30. |
| **JBI4** | Study subjects and setting described | Y when country, disease state, and design were extractable; U otherwise. |
| **JBI5** | Sufficient coverage of the identified/evaluable sample | Y when enhancing denominator equaled the total/evaluable sample; U/N when endpoint-specific denominator coverage was incomplete, depending on extent. |
| **JBI6** | Valid method for identifying progression | Y for RANO, Macdonald, or RANO/Macdonald; U for NR or nonstandard criteria. |
| **JBI7** | Standard/reliable spatial classification method | Y for dose/volume- or distance-based spatial definitions; U for descriptor/anatomic definitions; N for unclear spatial reference. |
| **JBI8** | Appropriate numerator/denominator analysis | Y when source numerators/denominators were manually confirmed/audited; U when source was present but not manually rechecked. |
| **JBI9** | Adequate imaging follow-up or response-rate handling | Y when imaging cadence was extractable; U/N when follow-up cadence or response-rate handling was incompletely reported. |

| **Source study/arm** | **Context** | **Design** | **JBI1** | **JBI2** | **JBI3** | **JBI4** | **JBI5** | **JBI6** | **JBI7** | **JBI8** | **JBI9** | **Overall** | **Key rationale** |
| --- | --- | --- | --- | --- | --- | --- | --- | --- | --- | --- | --- | --- | --- |
| **Ekinci et al. 2003** | Upfront first progression | Retrospective | U | U | N | Y | N | Y | U | Y | Y | High | mixed HGG/GBM population; retrospective or sampling approach not fully verifiable; small arm size (n=25); endpoint-specific evaluable denominator; descriptor/anatomic spatial definition |
| **Giese et al. 2004_A** | Upfront first progression | Clinical trial | Y | Y | N | Y | Y | Y | U | Y | Y | Moderate | small arm size (n=11); descriptor/anatomic spatial definition |
| **Giese et al. 2004_B** | Upfront first progression | Clinical trial | Y | Y | N | Y | Y | Y | U | Y | Y | Moderate | small arm size (n=13); descriptor/anatomic spatial definition |
| **Puchner et al. 2004** | Upfront first progression | Clinical trial | Y | Y | Y | Y | U | Y | U | Y | Y | Low | endpoint-specific evaluable denominator; descriptor/anatomic spatial definition |
| **Souhami et al. 2004_A** | Upfront first progression | Clinical trial | Y | Y | Y | Y | U | Y | N | Y | Y | Moderate | endpoint-specific evaluable denominator; spatial reference unclear |
| **Souhami et al. 2004_B** | Upfront first progression | Clinical trial | Y | Y | Y | Y | U | Y | N | Y | Y | Moderate | endpoint-specific evaluable denominator; spatial reference unclear |
| **Chang et al. 2007** | Upfront first progression | Retrospective | Y | U | U | Y | Y | Y | Y | Y | U | Moderate | retrospective or sampling approach not fully verifiable; modest arm size (n=48); imaging cadence/follow-up incompletely reported |
| **Park et al. 2007** | Upfront first progression | Retrospective | Y | U | N | Y | N | U | Y | Y | Y | High | retrospective or sampling approach not fully verifiable; small arm size (n=23); endpoint-specific evaluable denominator; progression criteria NR/other |
| **Showalter et al. 2007** | Upfront first progression | Retrospective | Y | U | Y | Y | U | U | U | Y | N | High | retrospective or sampling approach not fully verifiable; endpoint-specific evaluable denominator; progression criteria NR/other; descriptor/anatomic spatial definition; imaging cadence/follow-up incompletely reported |
| **Brandes et al. 2009** | Upfront first progression | Prospective | Y | Y | Y | Y | U | Y | U | Y | N | Moderate | endpoint-specific evaluable denominator; descriptor/anatomic spatial definition; imaging cadence/follow-up incompletely reported |
| **Panet-Raymond et al. 2009** | Upfront first progression | Retrospective | Y | U | U | Y | N | U | Y | Y | Y | Moderate | retrospective or sampling approach not fully verifiable; modest arm size (n=35); endpoint-specific evaluable denominator; progression criteria NR/other |
| **Tuettenberg et al. 2009** | Upfront first progression | Prospective | Y | Y | U | Y | Y | U | U | Y | Y | Moderate | modest arm size (n=32); progression criteria NR/other; descriptor/anatomic spatial definition |
| **Milano et al. 2010** | Upfront first progression | Retrospective | Y | U | Y | Y | U | Y | Y | Y | Y | Low | retrospective or sampling approach not fully verifiable; endpoint-specific evaluable denominator |
| **Minniti et al. 2010** | Upfront first progression | Retrospective | Y | U | Y | Y | U | Y | Y | Y | Y | Low | retrospective or sampling approach not fully verifiable; endpoint-specific evaluable denominator |
| **Chamberlain et al. 2011_A** | Upfront first progression | Retrospective | Y | U | Y | Y | Y | Y | U | Y | Y | Low | retrospective or sampling approach not fully verifiable; descriptor/anatomic spatial definition |
| **Dobelbower et al. 2011** | Upfront first progression | Retrospective | Y | U | N | Y | Y | Y | Y | Y | Y | Moderate | retrospective or sampling approach not fully verifiable; small arm size (n=20) |
| **McDonald et al. 2011** | Upfront first progression | Retrospective | Y | U | Y | Y | N | Y | Y | Y | Y | Moderate | retrospective or sampling approach not fully verifiable; endpoint-specific evaluable denominator |
| **Oh et al. 2011** | Upfront first progression | Retrospective | U | U | Y | Y | Y | U | Y | Y | U | Moderate | mixed HGG/GBM population; retrospective or sampling approach not fully verifiable; progression criteria NR/other; imaging cadence/follow-up incompletely reported |
| **Gunjur et al. 2012** | Upfront first progression | Retrospective | Y | U | Y | Y | N | Y | N | Y | N | High | retrospective or sampling approach not fully verifiable; endpoint-specific evaluable denominator; spatial reference unclear; imaging cadence/follow-up incompletely reported |
| **Konishi et al. 2012** | Upfront first progression | Retrospective | Y | U | U | Y | U | Y | Y | Y | Y | Moderate | retrospective or sampling approach not fully verifiable; modest arm size (n=43); endpoint-specific evaluable denominator |
| **Monjazeb et al. 2012** | Upfront first progression | Prospective | Y | Y | N | Y | U | U | Y | Y | Y | Moderate | small arm size (n=21); endpoint-specific evaluable denominator; progression criteria NR/other |
| **Niyazi et al. 2012** | Upfront first progression | Retrospective | Y | U | Y | Y | N | Y | Y | Y | Y | Moderate | retrospective or sampling approach not fully verifiable; endpoint-specific evaluable denominator |
| **Piroth et al. 2012** | Upfront first progression | Prospective | Y | Y | N | Y | U | Y | Y | Y | Y | Moderate | small arm size (n=22); endpoint-specific evaluable denominator |
| **De Bonis et al. 2013** | Upfront first progression | Retrospective | Y | U | Y | Y | U | Y | Y | Y | Y | Low | retrospective or sampling approach not fully verifiable; endpoint-specific evaluable denominator |
| **Dorner et al. 2013** | Upfront first progression | Prospective | U | Y | N | Y | Y | U | U | Y | Y | Moderate | mixed HGG/GBM population; small arm size (n=19); progression criteria NR/other; descriptor/anatomic spatial definition |
| **Ho et al. 2013** | Upfront first progression | Retrospective | Y | U | Y | Y | N | Y | N | Y | Y | Moderate | retrospective or sampling approach not fully verifiable; endpoint-specific evaluable denominator; spatial reference unclear |
| **Kimura et al. 2013** | Upfront first progression | Retrospective | Y | U | U | Y | Y | U | U | Y | Y | Moderate | retrospective or sampling approach not fully verifiable; modest arm size (n=49); progression criteria NR/other; descriptor/anatomic spatial definition |
| **Ogura et al. 2013** | Upfront first progression | Retrospective | Y | U | U | Y | N | Y | Y | Y | Y | Moderate | retrospective or sampling approach not fully verifiable; modest arm size (n=37); endpoint-specific evaluable denominator |
| **Petrecca et al. 2013** | Upfront first progression | Retrospective | Y | U | N | Y | Y | Y | U | Y | U | Moderate | retrospective or sampling approach not fully verifiable; small arm size (n=20); descriptor/anatomic spatial definition; imaging cadence/follow-up incompletely reported |
| **Sherriff et al. 2013** | Upfront first progression | Retrospective | Y | U | Y | Y | N | Y | Y | Y | Y | Moderate | retrospective or sampling approach not fully verifiable; endpoint-specific evaluable denominator |
| **Shields et al. 2013** | Upfront first progression | Retrospective | Y | U | N | Y | N | Y | Y | Y | Y | Moderate | retrospective or sampling approach not fully verifiable; small arm size (n=23); endpoint-specific evaluable denominator |
| **Adeberg et al. 2014** | Upfront first progression | Retrospective | Y | U | Y | Y | N | U | U | Y | Y | Moderate | retrospective or sampling approach not fully verifiable; endpoint-specific evaluable denominator; progression criteria NR/other; descriptor/anatomic spatial definition |
| **Iuchi et al. 2014** | Upfront first progression | Clinical trial | Y | Y | U | Y | N | U | Y | Y | Y | Moderate | modest arm size (n=46); endpoint-specific evaluable denominator; progression criteria NR/other |
| **Miwa et al. 2014** | Upfront first progression | Prospective | Y | Y | U | Y | N | U | Y | Y | N | High | modest arm size (n=45); endpoint-specific evaluable denominator; progression criteria NR/other; imaging cadence/follow-up incompletely reported |
| **Omuro et al. 2014** | Upfront first progression | Clinical trial | Y | Y | U | Y | U | Y | N | Y | Y | Moderate | modest arm size (n=40); endpoint-specific evaluable denominator; spatial reference unclear |
| **Reddy et al. 2014** | Upfront first progression | Clinical trial | Y | Y | N | Y | U | Y | N | Y | Y | Moderate | small arm size (n=24); endpoint-specific evaluable denominator; spatial reference unclear |
| **Tejada et al. 2014** | Upfront first progression | Retrospective | Y | U | Y | Y | U | Y | U | Y | Y | Moderate | retrospective or sampling approach not fully verifiable; endpoint-specific evaluable denominator; descriptor/anatomic spatial definition |
| **Chen et al. 2015** | Upfront first progression | Retrospective | Y | U | Y | Y | Y | U | Y | Y | U | Moderate | retrospective or sampling approach not fully verifiable; progression criteria NR/other; imaging cadence/follow-up incompletely reported |
| **Elson et al. 2015** | Upfront first progression | Retrospective | Y | U | Y | Y | N | U | Y | Y | Y | Moderate | retrospective or sampling approach not fully verifiable; endpoint-specific evaluable denominator; progression criteria NR/other |
| **Nestler et al. 2015** | Upfront first progression | Post hoc | Y | Y | Y | Y | Y | U | U | Y | U | Moderate | progression criteria NR/other; descriptor/anatomic spatial definition; imaging cadence/follow-up incompletely reported |
| **Ney et al. 2015** | Upfront first progression | Clinical trial | Y | Y | U | Y | U | Y | Y | Y | Y | Low | modest arm size (n=30); endpoint-specific evaluable denominator |
| **Thiepold et al. 2015_A** | Upfront first progression | Retrospective | Y | U | U | Y | U | U | U | Y | N | High | retrospective or sampling approach not fully verifiable; modest arm size (n=46); endpoint-specific evaluable denominator; progression criteria NR/other; descriptor/anatomic spatial definition; imaging cadence/follow-up incompletely reported |
| **Thiepold et al. 2015_B** | Upfront first progression | Retrospective | Y | U | U | Y | U | U | U | Y | N | High | retrospective or sampling approach not fully verifiable; modest arm size (n=46); endpoint-specific evaluable denominator; progression criteria NR/other; descriptor/anatomic spatial definition; imaging cadence/follow-up incompletely reported |
| **Buglione et al. 2016** | Upfront first progression | Retrospective | Y | U | Y | Y | N | Y | Y | Y | N | Moderate | retrospective or sampling approach not fully verifiable; endpoint-specific evaluable denominator; imaging cadence/follow-up incompletely reported |
| **Harat et al. 2016** | Upfront first progression | Prospective | Y | Y | U | Y | N | U | Y | Y | Y | Moderate | modest arm size (n=34); endpoint-specific evaluable denominator; progression criteria NR/other |
| **Wick et al. 2016_A** | Upfront first progression | Clinical trial | Y | Y | Y | Y | N | Y | U | Y | Y | Moderate | endpoint-specific evaluable denominator; descriptor/anatomic spatial definition |
| **Wick et al. 2016_B** | Upfront first progression | Clinical trial | Y | Y | Y | Y | U | Y | U | Y | Y | Low | endpoint-specific evaluable denominator; descriptor/anatomic spatial definition |
| **Lundemann et al. 2017** | Upfront first progression | Retrospective | Y | U | Y | Y | U | Y | Y | Y | Y | Low | retrospective or sampling approach not fully verifiable; endpoint-specific evaluable denominator |
| **Pessina et al. 2017** | Upfront first progression | Retrospective | Y | U | Y | Y | U | U | N | Y | N | High | retrospective or sampling approach not fully verifiable; endpoint-specific evaluable denominator; progression criteria NR/other; spatial reference unclear; imaging cadence/follow-up incompletely reported |
| **Rapp et al. 2017** | Upfront first progression | Retrospective | Y | U | Y | Y | U | Y | Y | Y | Y | Low | retrospective or sampling approach not fully verifiable; endpoint-specific evaluable denominator |
| **Yan et al. 2017** | Upfront first progression | Retrospective | Y | U | U | Y | Y | U | U | Y | U | Moderate | retrospective or sampling approach not fully verifiable; modest arm size (n=31); progression criteria NR/other; descriptor/anatomic spatial definition; imaging cadence/follow-up incompletely reported |
| **Bette et al. 2018** | Upfront first progression | Retrospective | Y | U | Y | Y | Y | Y | U | Y | U | Moderate | retrospective or sampling approach not fully verifiable; descriptor/anatomic spatial definition; imaging cadence/follow-up incompletely reported |
| **Jayamanne et al. 2018** | Upfront first progression | Retrospective | Y | U | Y | Y | U | Y | Y | Y | Y | Low | retrospective or sampling approach not fully verifiable; endpoint-specific evaluable denominator |
| **Rowe et al. 2018** | Upfront first progression | Retrospective | Y | U | Y | Y | U | Y | N | Y | Y | Moderate | retrospective or sampling approach not fully verifiable; endpoint-specific evaluable denominator; spatial reference unclear |
| **Schaub et al. 2018** | Upfront first progression | Post hoc | Y | Y | Y | Y | Y | U | U | Y | Y | Low | progression criteria NR/other; descriptor/anatomic spatial definition |
| **Kim et al. 2019** | Upfront first progression | Retrospective | Y | U | Y | Y | U | U | Y | Y | Y | Moderate | retrospective or sampling approach not fully verifiable; endpoint-specific evaluable denominator; progression criteria NR/other |
| **Sheu et al. 2019** | Upfront first progression | Retrospective | Y | U | Y | Y | U | U | N | Y | Y | Moderate | retrospective or sampling approach not fully verifiable; endpoint-specific evaluable denominator; progression criteria NR/other; spatial reference unclear |
| **Faustino et al. 2020** | Upfront first progression | Retrospective | Y | U | Y | Y | U | U | Y | Y | Y | Moderate | retrospective or sampling approach not fully verifiable; endpoint-specific evaluable denominator; progression criteria NR/other |
| **Fleischmann et al. 2020** | Upfront first progression | Retrospective | Y | U | U | Y | Y | Y | Y | Y | U | Moderate | retrospective or sampling approach not fully verifiable; modest arm size (n=36); imaging cadence/follow-up incompletely reported |
| **Jiang et al. 2020** | Upfront first progression | Retrospective | Y | U | Y | Y | Y | Y | U | Y | Y | Low | retrospective or sampling approach not fully verifiable; descriptor/anatomic spatial definition |
| **Kazerooni et al. 2020** | Upfront first progression | Retrospective | Y | U | Y | Y | Y | Y | U | Y | U | Moderate | retrospective or sampling approach not fully verifiable; descriptor/anatomic spatial definition; imaging cadence/follow-up incompletely reported |
| **Sarria et al. 2020** | Upfront first progression | Retrospective | Y | U | Y | Y | U | Y | Y | Y | Y | Low | retrospective or sampling approach not fully verifiable; endpoint-specific evaluable denominator |
| **Yamaki et al. 2020** | Upfront first progression | Retrospective | Y | U | Y | Y | U | U | U | Y | N | High | retrospective or sampling approach not fully verifiable; endpoint-specific evaluable denominator; progression criteria NR/other; descriptor/anatomic spatial definition; imaging cadence/follow-up incompletely reported |
| **Comas et al. 2021** | Upfront first progression | Retrospective | Y | U | Y | Y | U | Y | Y | Y | Y | Low | retrospective or sampling approach not fully verifiable; endpoint-specific evaluable denominator |
| **Hirono et al. 2021_A** | Upfront first progression | Retrospective | Y | U | N | Y | U | Y | U | Y | Y | Moderate | retrospective or sampling approach not fully verifiable; small arm size (n=23); endpoint-specific evaluable denominator; descriptor/anatomic spatial definition |
| **Hirono et al. 2021_B** | Upfront first progression | Retrospective | Y | U | N | Y | N | Y | U | Y | Y | High | retrospective or sampling approach not fully verifiable; small arm size (n=7); endpoint-specific evaluable denominator; descriptor/anatomic spatial definition |
| **Kim et al. 2021** | Upfront first progression | Clinical trial | Y | Y | N | Y | N | Y | Y | Y | Y | Moderate | small arm size (n=23); endpoint-specific evaluable denominator |
| **Shim et al. 2021** | Upfront first progression | Retrospective | Y | U | Y | Y | Y | Y | U | Y | Y | Low | retrospective or sampling approach not fully verifiable; descriptor/anatomic spatial definition |
| **Ali et al. 2022** | Upfront first progression | Clinical trial | Y | Y | U | Y | U | Y | Y | Y | Y | Low | modest arm size (n=30); endpoint-specific evaluable denominator |
| **Glas et al. 2022_A** | Upfront first progression | Post hoc | Y | Y | Y | Y | Y | U | Y | Y | Y | Low | progression criteria NR/other |
| **Glas et al. 2022_B** | Upfront first progression | Post hoc | Y | Y | Y | Y | Y | U | Y | Y | Y | Low | progression criteria NR/other |
| **Yoo et al. 2022_A** | Upfront first progression | Retrospective | Y | U | U | Y | N | Y | Y | Y | N | High | retrospective or sampling approach not fully verifiable; modest arm size (n=41); endpoint-specific evaluable denominator; imaging cadence/follow-up incompletely reported |
| **Yoo et al. 2022_B** | Upfront first progression | Retrospective | Y | U | Y | Y | U | Y | Y | Y | N | Moderate | retrospective or sampling approach not fully verifiable; endpoint-specific evaluable denominator; imaging cadence/follow-up incompletely reported |
| **Yoo et al. 2022_C** | Upfront first progression | Retrospective | Y | U | Y | Y | U | Y | Y | Y | N | Moderate | retrospective or sampling approach not fully verifiable; endpoint-specific evaluable denominator; imaging cadence/follow-up incompletely reported |
| **Demircan et al. 2023** | Upfront first progression | Retrospective | Y | U | Y | Y | U | Y | Y | Y | N | Moderate | retrospective or sampling approach not fully verifiable; endpoint-specific evaluable denominator; imaging cadence/follow-up incompletely reported |
| **Guberina et al. 2023** | Upfront first progression | Retrospective | Y | U | Y | Y | Y | Y | Y | Y | U | Low | retrospective or sampling approach not fully verifiable; imaging cadence/follow-up incompletely reported |
| **Liu et al. 2023** | Upfront first progression | Retrospective | Y | U | Y | Y | U | Y | Y | Y | Y | Low | retrospective or sampling approach not fully verifiable; endpoint-specific evaluable denominator |
| **Mendoza et al. 2023** | Upfront first progression | Prospective | Y | Y | U | Y | U | Y | Y | Y | Y | Low | modest arm size (n=30); endpoint-specific evaluable denominator |
| **Minniti et al. 2023** | Upfront first progression | Retrospective | Y | U | Y | Y | U | Y | Y | Y | Y | Low | retrospective or sampling approach not fully verifiable; endpoint-specific evaluable denominator |
| **Mizuhata et al. 2023_A** | Upfront first progression | Retrospective | Y | U | U | Y | Y | Y | U | Y | U | Moderate | retrospective or sampling approach not fully verifiable; modest arm size (n=34); descriptor/anatomic spatial definition; imaging cadence/follow-up incompletely reported |
| **Mizuhata et al. 2023_B** | Upfront first progression | Retrospective | Y | U | N | Y | Y | Y | U | Y | U | Moderate | retrospective or sampling approach not fully verifiable; small arm size (n=24); descriptor/anatomic spatial definition; imaging cadence/follow-up incompletely reported |
| **Noeuveglise et al. 2023** | Upfront first progression | Retrospective | Y | U | Y | Y | Y | U | U | Y | Y | Moderate | retrospective or sampling approach not fully verifiable; progression criteria NR/other; descriptor/anatomic spatial definition |
| **Di Perri et al. 2024** | Upfront first progression | Retrospective | Y | U | Y | Y | N | Y | Y | Y | Y | Moderate | retrospective or sampling approach not fully verifiable; endpoint-specific evaluable denominator |
| **Senyurek et al. 2024** | Upfront first progression | Retrospective | Y | U | Y | Y | U | Y | Y | Y | N | Moderate | retrospective or sampling approach not fully verifiable; endpoint-specific evaluable denominator; imaging cadence/follow-up incompletely reported |
| **Toyoda et al. 2024_A** | Upfront first progression | Retrospective | Y | U | Y | Y | U | Y | U | Y | Y | Moderate | retrospective or sampling approach not fully verifiable; endpoint-specific evaluable denominator; descriptor/anatomic spatial definition |
| **Braschi et al. 2025** | Upfront first progression | Retrospective | Y | U | Y | Y | Y | Y | Y | Y | Y | Low | retrospective or sampling approach not fully verifiable |
| **Crompton et al. 2025_A** | Upfront first progression | Retrospective | Y | U | Y | Y | U | U | Y | Y | N | Moderate | retrospective or sampling approach not fully verifiable; endpoint-specific evaluable denominator; progression criteria NR/other; imaging cadence/follow-up incompletely reported |
| **Crompton et al. 2025_B** | Upfront first progression | Retrospective | Y | U | Y | Y | U | U | Y | Y | N | Moderate | retrospective or sampling approach not fully verifiable; endpoint-specific evaluable denominator; progression criteria NR/other; imaging cadence/follow-up incompletely reported |
| **Fujimoto et al. 2025_A** | Upfront first progression | Retrospective | Y | U | U | Y | U | Y | U | Y | Y | Moderate | retrospective or sampling approach not fully verifiable; modest arm size (n=44); endpoint-specific evaluable denominator; descriptor/anatomic spatial definition |
| **Fujimoto et al. 2025_B** | Upfront first progression | Retrospective | Y | U | Y | Y | Y | Y | U | Y | Y | Low | retrospective or sampling approach not fully verifiable; descriptor/anatomic spatial definition |
| **Laviv et al. 2025** | Upfront first progression | Retrospective | Y | U | Y | Y | Y | Y | U | Y | U | Moderate | retrospective or sampling approach not fully verifiable; descriptor/anatomic spatial definition; imaging cadence/follow-up incompletely reported |
| **Moore-Palhares et al. 2025** | Upfront first progression | Prospective | Y | Y | Y | Y | U | Y | Y | Y | Y | Low | endpoint-specific evaluable denominator |
| **Takido et al. 2025** | Upfront first progression | Retrospective | Y | U | N | Y | U | U | U | Y | N | High | retrospective or sampling approach not fully verifiable; small arm size (n=21); endpoint-specific evaluable denominator; progression criteria NR/other; descriptor/anatomic spatial definition; imaging cadence/follow-up incompletely reported |
| **Tian et al. 2025** | Upfront first progression | Retrospective | Y | U | U | Y | U | Y | Y | Y | N | Moderate | retrospective or sampling approach not fully verifiable; modest arm size (n=41); endpoint-specific evaluable denominator; imaging cadence/follow-up incompletely reported |
| **Matsuyama et al. 2026** | Upfront first progression | Retrospective | Y | U | Y | Y | N | Y | Y | Y | Y | Moderate | retrospective or sampling approach not fully verifiable; endpoint-specific evaluable denominator |
| **Norden et al. 2008** | Post-BEV failure | Retrospective | U | U | N | Y | U | Y | U | Y | Y | High | mixed HGG/GBM population; retrospective or sampling approach not fully verifiable; small arm size (n=26); endpoint-specific evaluable denominator; descriptor/anatomic spatial definition |
| **Iwamoto et al. 2009** | Post-BEV failure | Retrospective | Y | U | U | Y | U | Y | U | Y | N | High | retrospective or sampling approach not fully verifiable; modest arm size (n=37); endpoint-specific evaluable denominator; descriptor/anatomic spatial definition; imaging cadence/follow-up incompletely reported |
| **Chamberlain et al. 2011_B** | Post-BEV failure | Retrospective | Y | U | Y | Y | Y | Y | U | Y | Y | Low | retrospective or sampling approach not fully verifiable; descriptor/anatomic spatial definition |
| **Pope et al. 2011_A** | Post-BEV failure | Post hoc | Y | Y | Y | Y | U | Y | Y | Y | Y | Low | endpoint-specific evaluable denominator |
| **Pope et al. 2011_B** | Post-BEV failure | Post hoc | Y | Y | Y | Y | N | Y | Y | Y | Y | Low | endpoint-specific evaluable denominator |
| **Desjardins et al. 2012** | Post-BEV failure | Clinical trial | Y | Y | U | Y | N | Y | U | Y | Y | Moderate | modest arm size (n=32); endpoint-specific evaluable denominator; descriptor/anatomic spatial definition |
| **Perez-Larraya et al. 2012** | Post-BEV failure | Retrospective | Y | U | Y | Y | U | Y | N | Y | Y | Moderate | retrospective or sampling approach not fully verifiable; endpoint-specific evaluable denominator; spatial reference unclear |
| **Bahr et al. 2014** | Post-BEV failure | Mixed | Y | U | Y | Y | U | Y | U | Y | N | Moderate | retrospective or sampling approach not fully verifiable; endpoint-specific evaluable denominator; descriptor/anatomic spatial definition; imaging cadence/follow-up incompletely reported |
| **Soffietti et al. 2014** | Post-BEV failure | Clinical trial | Y | Y | U | Y | Y | Y | U | Y | U | Moderate | modest arm size (n=42); descriptor/anatomic spatial definition; imaging cadence/follow-up incompletely reported |
| **Mamo et al. 2016** | Post-BEV failure | Retrospective | Y | U | U | Y | Y | U | U | Y | U | Moderate | retrospective or sampling approach not fully verifiable; modest arm size (n=34); progression criteria NR/other; descriptor/anatomic spatial definition; imaging cadence/follow-up incompletely reported |
| **Cachia et al. 2017** | Post-BEV failure | Retrospective | Y | U | Y | Y | Y | Y | U | Y | U | Moderate | retrospective or sampling approach not fully verifiable; descriptor/anatomic spatial definition; imaging cadence/follow-up incompletely reported |
| **Park et al. 2020** | Post-BEV failure | Retrospective | Y | U | Y | Y | U | Y | Y | Y | Y | Low | retrospective or sampling approach not fully verifiable; endpoint-specific evaluable denominator |
| **Ciammella et al. 2013** | Post-reRT failure | Retrospective | Y | U | Y | Y | U | U | N | Y | Y | Moderate | retrospective or sampling approach not fully verifiable; endpoint-specific evaluable denominator; progression criteria NR/other; spatial reference unclear |
| **Niyazi et al. 2014** | Post-reRT failure | Retrospective | U | U | U | Y | Y | Y | Y | Y | Y | Moderate | mixed HGG/GBM population; retrospective or sampling approach not fully verifiable; modest arm size (n=31) |
| **Straube et al. 2017** | Post-reRT failure | Retrospective | Y | U | N | Y | U | Y | Y | Y | Y | Moderate | retrospective or sampling approach not fully verifiable; small arm size (n=26); endpoint-specific evaluable denominator |
| **Ene et al. 2019** | Post-reRT failure | Retrospective | U | U | U | Y | Y | Y | Y | Y | Y | Moderate | mixed HGG/GBM population; retrospective or sampling approach not fully verifiable; modest arm size (n=47) |
| **Dono et al. 2021** | Post-reRT failure | Retrospective | Y | U | U | Y | U | Y | N | Y | Y | Moderate | retrospective or sampling approach not fully verifiable; modest arm size (n=43); endpoint-specific evaluable denominator; spatial reference unclear |
| **Datta et al. 2023** | Post-reRT failure | Retrospective | U | U | U | Y | Y | U | Y | Y | Y | Moderate | mixed HGG/GBM population; retrospective or sampling approach not fully verifiable; modest arm size (n=37); progression criteria NR/other |
| **You et al. 2023_A** | Post-reRT failure | Retrospective | Y | U | U | Y | Y | Y | N | Y | Y | Moderate | retrospective or sampling approach not fully verifiable; modest arm size (n=35); spatial reference unclear |
| **You et al. 2023_B** | Post-reRT failure | Retrospective | Y | U | N | Y | Y | Y | N | Y | Y | Moderate | retrospective or sampling approach not fully verifiable; small arm size (n=29); spatial reference unclear |
| **Christ et al. 2024** | Post-reRT failure | Retrospective | Y | U | Y | Y | N | Y | N | Y | N | High | retrospective or sampling approach not fully verifiable; endpoint-specific evaluable denominator; spatial reference unclear; imaging cadence/follow-up incompletely reported |
| **Pepper et al. 2024** | Post-reRT failure | Retrospective | Y | U | Y | Y | N | Y | Y | Y | Y | Moderate | retrospective or sampling approach not fully verifiable; endpoint-specific evaluable denominator |
| **Rogers et al. 2024** | Post-reRT failure | Retrospective | U | U | U | Y | N | U | Y | Y | Y | High | mixed HGG/GBM population; retrospective or sampling approach not fully verifiable; modest arm size (n=47); endpoint-specific evaluable denominator; progression criteria NR/other |
| **Tong et al. 2024** | Post-reRT failure | Retrospective | Y | U | Y | Y | U | U | Y | Y | N | Moderate | retrospective or sampling approach not fully verifiable; endpoint-specific evaluable denominator; progression criteria NR/other; imaging cadence/follow-up incompletely reported |
| **Margulies et al. 2025** | Post-reRT failure | Retrospective | Y | U | Y | Y | Y | Y | Y | Y | Y | Low | retrospective or sampling approach not fully verifiable |
| **Dorner et al. 2013_B** | Post-other salvage failure | Prospective | U | Y | N | Y | Y | U | U | Y | Y | Moderate | mixed HGG/GBM population; small arm size (n=22); progression criteria NR/other; descriptor/anatomic spatial definition |
| **Moon et al. 2022_B** | Post-other salvage failure | Retrospective | Y | U | N | Y | U | Y | N | Y | Y | High | retrospective or sampling approach not fully verifiable; small arm size (n=19); endpoint-specific evaluable denominator; spatial reference unclear |

Tool basis: JBI Critical Appraisal Checklist for Studies Reporting Prevalence Data; Munn Z et al., Int J Evid Based Healthc. 2015;13:147-153.

**Supplementary Figure 1.** Random-effects forest plots of enhancing involvement (marginal and broad escape) at first progression after upfront RT/TMZ±TTF.

**Marginal**

**Broad escape**

**Supplementary Figure 2.** Random-effects forest plots of enhancing involvement (marginal and broad escape pattern) post-salvage progression (≥2nd progression).

**Marginal**

**Broad escape**

**Supplementary results**

**Robustness and leave-one-out diagnostics**

Influence and leave-one-out analyses suggested that no single arm materially drove the primary pooled estimates. For upfront first-progression local enhancing involvement (k=94 arms), leave-one-out re-estimation varied narrowly from 0.794 to 0.803 (range 0.0083), supporting stability of the summary effect to individual-study removal. For salvage-treated cohorts at subsequent progression (k=27 arms), the pooled local estimate varied from 0.589 to 0.618 (range 0.0288). The slightly wider range relative to upfront cohorts is consistent with the smaller evidence base and greater between-study variability in salvage settings, but conclusions were unchanged. The broad escape-pattern progression endpoint was similarly robust: in upfront cohorts the pooled estimate varied from 0.246 to 0.253 (range 0.0074), and in salvage cohorts from 0.366 to 0.394 (range 0.0279). Overall, these analyses indicate that the principal pooled estimates were not dominated by any single study.

**Small-study effects**

We assessed small-study effects using linear regression tests of funnel plot asymmetry (Egger-type) for endpoints with k≥10. In upfront first-progression cohorts, there was evidence of asymmetry for local (p=0.0141), distant (p=0.0026), and marginal enhancing involvement (p<0.0001), but not for broad escape-pattern progression endpoint (p=0.0660). In salvage-treated cohorts overall, asymmetry was detected for distant involvement (p<0.0001), whereas tests were not significant for local (p=0.4788), non-local (p=0.4431), or broad escape-pattern progression (p=0.4667). Given the substantial between-study heterogeneity and selective reporting for certain endpoints, especially marginal involvement, these asymmetry tests were interpreted as sensitivity analyses rather than definitive evidence of publication bias.

**Heterogeneity**

Across outcomes, heterogeneity remained substantial, consistent with clinical and methodological diversity across included studies. For first progression after RT/TMZ±TTF, heterogeneity was high for local involvement (I²=84.8%, τ²=0.702 on the logit scale), distant involvement (I²=85.2%, τ²=0.635), marginal involvement (I²=84.0%, τ²=0.701), and the broad escape-pattern progression endpoint (I²=83.8%, τ²=0.525). For salvage-treated cohorts at subsequent progression, heterogeneity remained high for local involvement (I²=80.5%, τ²=0.392), distant involvement (I²=75.5%, τ²=0.326), marginal involvement (I²=91.6%, τ²=0.907), and broad escape-pattern progression (I²=80.1%, τ²=0.439). For nonenhancing/FLAIR-dominant events, heterogeneity remained substantial in recurrent/salvage cohorts (I²=75.7%, τ²=0.595). These findings indicate that between-study differences exceeded within-study sampling error, which is common in proportion meta-analyses and is amplified when operational definitions vary, denominators differ, and reporting is selective.

**Exploratory heterogeneity assessment by meta-regression**

Univariable meta-regression models were used to explore candidate moderators of local enhancing involvement and broad escape-pattern progression and should be interpreted as exploratory. In upfront local models, the largest reductions in between-study variance were seen for progression criteria (pseudo-R² on τ²=9.3% on the analyzable subset), study design (8.5%), and spatial reference framework (5.4%), whereas publication year, denominator relation, recurrence framework, and mixed-HGG status did not meaningfully reduce τ² despite several nominally significant omnibus tests. In salvage local models, mixed-HGG status showed the clearest signal (omnibus p=0.0072; pseudo-R²=8.5%), while other moderators explained little residual variance. For the broad escape-pattern progression endpoint, study design produced the largest τ² reduction in upfront cohorts (pseudo-R²=9.8%), whereas in salvage cohorts the strongest exploratory signals were mixed-HGG status (pseudo-R²=15.3%; omnibus p=0.0015) and publication year (pseudo-R²=14.8%; omnibus p=0.0029). Overall, residual heterogeneity remained substantial across models, indicating that the routinely extractable workbook variables explained only a limited fraction of between-study variability.

**Supplementary Figure 3.** Trim-and-fill-adjusted funnel plots for local, marginal, distant, and broad-escape enhancing involvement (k ≥ 10). The top row shows first progression after upfront RT/TMZ ± TTF (A, local; B, distant; C, marginal; D, escape), whereas the bottom row shows subsequent progression after salvage therapy (E, local; F, distant; G, escape).

A

B

C

D

E

F

G
